# Bridging cognitive reserve and cerebellar networks: counteracting brain damage in patients with Alzheimer’s disease at different clinical stages

**DOI:** 10.3389/fncel.2026.1716783

**Published:** 2026-02-06

**Authors:** Laura Serra, Martina Rizzuti, Sabrina Bonarota, Giulia Caruso, Carlotta Di Domenico, Matteo Mancini, Federico Giove, Carlo Caltagirone, Francesca Gelfo, Laura Petrosini

**Affiliations:** 1 Department of Human Sciences, Università degli Studi Guglielmo Marconi, Rome, Italy; 2 Neuroimaging Laboratory, Fondazione Santa Lucia, Rome, Italy; 3 Laboratory of Experimental and Behavioral Neurophysiology, Fondazione Santa Lucia, Rome, Italy; 4 Department of Psychology, Sapienza University of Rome, Rome, Italy; 5 Museo Storico della Fisica e Centro Studi e Ricerche Enrico Fermi, Rome, Italy; 6 Scientific Direction, Fondazione Santa Lucia IRCCS, Rome, Italy

**Keywords:** Alzheimer’s disease, cerebellar cognitive reserve, cerebellar-cortical networks, cerebellum, cognitive reserve, source-based morphometry, structural connectivity

## Abstract

**Introduction:**

Alzheimer’s disease (AD) is a progressive neurodegenerative condition characterized by cognitive decline and brain atrophy. Recent evidence shows that the cerebellum also undergoes structural and functional alterations. The concept of cognitive reserve (CR) explains individual resilience to brain pathology, while the hypothesis of cerebellar cognitive reserve (CbCR) sustains an enhanced activity in cerebellar networks at rest and a more efficient recruitment of accessory areas during cognitive tasks. This study investigated structural connectivity changes in cerebellar-cortical networks across the AD continuum.

**Methods:**

A total of 179 participants were enrolled, encompassing 46 AD patients, 51 with amnestic mild cognitive impairment (aMCI), 46 with subjective cognitive decline (SCD), and 36 healthy subjects (HS). CR was defined by years of formal education. Whole-brain T1-weighted images were analysed using source-based morphometry (SBM) to identify patterns of grey matter concentration (GMC) covariance.

**Results:**

Three main networks were identified: the cerebellum-basal ganglia-cingulum (CBGC), the anterior cerebellum-supplementary motor arearetrosplenial cortex (ACSMARC), and the posterior cerebellum-orbitofrontal cortex (PCOC), with the cerebellum as the only common structure. All networks showed progressive reductions in GMC covariance along the clinical continuum, with a specific pattern of GMC reduction according to diagnostic groups. Importantly, CbCR modulated connectivity within all networks, with higher levels associated with preserved structural integrity and better cognitive outcomes.

**Discussion:**

These findings provide evidence of progressive cerebellar-cortical disconnection in AD pathology and highlight cerebellar reserve as a potential protective factor, suggesting that the CbCR assessment and specific interventions tailored to the cerebellar cognitive functions may help delay the cognitive decline.

## 1 Introduction

Alzheimer’s disease (AD), the most common cause of cognitive decline in Western populations (Ferri et al., 2005), is a progressive neurodegenerative disease characterized by brain atrophy, particularly in the temporal, parietal, and frontal lobes, resulting in memory disorders, difficulties with language, executive, and visuospatial functions (Knopman et al., 2021). The AD preclinical phase is typically asymptomatic or limited to subjective cognitive decline (SCD), in which individuals perceive a decline in cognitive abilities despite normal test performances (Jessen et al., 2020). The AD early symptomatic phase is known as amnestic mild cognitive impairment (aMCI), in which memory deficits become objectively measurable, even if they do not yet interfere with daily life. At this stage, neurodegeneration often begins in medial temporal structures, like the entorhinal cortex and hippocampus. In the AD later phase, brain atrophy extends to lateral temporal, parietal, and frontal association areas, and the resulting cognitive impairment disrupts daily functioning (Braak and Braak, 1996).

While traditionally underexplored in AD, recent evidence suggests the cerebellum may also be affected (Jacobs et al., 2018; Liang and Carlson, 2020; Yang et al., 2024; Chen et al., 2024; Samstag et al., 2025). While structural neuroimaging revealed cerebellar grey matter (GM) atrophy as early as the MCI stage, beginning in the vermian and paravermian (I–VI lobules), and later involving posterior lobules (Toniolo et al., 2018), functional neuroimaging showed disrupted cerebellar-cortical connectivity (Bai et al., 2011; Olivito et al., 2020; Zhou et al., 2021; Wang et al., 2021). Novel neuropathological evidence reports that individuals with lower postmortem cerebellar weights showed more cognitive decline, independently of classical AD neuropathology markers, suggesting a cerebellar role in dementia (Samstag et al., 2025).

The individual differences in the vulnerability of the cerebral cortex to aging processes or pathological damage have been explained by introducing the concept of reserve (Stern, 2012; Stern et al., 2020) with its three forms, namely brain, cognitive, and neural reserve (although the three forms of reserve have been encompassed in the usual label cognitive reserve, CR): brain reserve, which is mainly morphological and quantitative in nature, refers to structural characteristics, such as the number of neurons and synapses (Stern, 2012; Stern et al., 2020) Cognitive reserve concerns the use of already acquired mental strategies or the adoption of compensatory mechanisms to cope with functional loss (Stern, 2012; Barulli and Stern, 2013; Cabeza et al., 2018; Stern et al., 2020). Finally, neural reserve is linked to the functionality of brain networks and their ability to process information efficiently (Stern, 2012; Serra et al., 2017; Serra et al., 2018). The reserves are retained to provide compensatory mechanisms against cognitive decline (Stern, 2012; Serra et al., 2018).

Specific indicators of experiential enrichment, such as level of education, career complexity, engagement in recreational activities, and cognitive performance, have traditionally been used as indirect measures of CR (Stern, 2012; Rentz et al., 2010; Arenaza-Urquijo et al., 2017; Serra et al., 2018). By definition, CR is a latent and unobservable construct and therefore must be operationalized through proxy measures, among them, the years of formal education have been consistently identified as one of the most robust and reliable indicators. Since the seminal work of Stern (2002, 2009), education has been widely adopted as a proxy of CR across epidemiological, clinical, and neuroimaging studies, reflecting early-life intellectual enrichment and sustained cognitive engagement. Large-scale cohort studies, meta-analyses, and umbrella reviews have shown that education is typically used as the sole proxy of cognitive reserve and is strongly associated with delayed clinical manifestation of neurodegenerative diseases, reduced cognitive decline, and greater resilience to brain pathology (Barulli and Stern, 2013; Stern et al., 2018; Wilson et al., 2019; Lövdén et al., 2020; Seyedsalehi et al., 2023; Pinto et al., 2024). Importantly, while CR is inherently multifactorial, the use of education as a single proxy does not imply a reductionist interpretation of the construct; rather, it represents a theoretically grounded and methodologically pragmatic choice that ensures standardization, reproducibility, and comparability across studies.

Several structural MRI studies using the voxel-based morphometry approach (VBM) or cortical complexity techniques have demonstrated that individuals with higher CR can tolerate greater brain atrophy before exhibiting clinical symptoms of AD (Groot et al., 2018; Zhu et al., 2021; Serra et al., 2011; Querbes et al., 2009; Cai et al., 2025). Moreover, resting-state functional MRI studies revealed that participants with higher CR exhibit stronger functional connectivity within networks critical for memory and executive functions, such as the default mode network and fronto-parietal circuits, and show an increased capacity to recruit alternative neuronal resources (Bozzali et al., 2015; Serra et al., 2016; Weiler et al., 2018; Wook Yoo et al., 2015). These findings suggest that CR not only supports brain structural integrity but also enhances network functional resilience, thereby contributing to improved clinical outcomes.

Unaccountably, only a few studies have directly addressed the cerebellar role within the reserve model in clinical contexts. Serra et al. (2017) showed that the volume of cerebellar GM was positively correlated with CR indices, especially in crus I and lobules IV and VII, in both HS and aMCI patients. Moreover, Serra et al. (2016) observed a reduction in resting-state functional connectivity in a network comprising frontal, temporal, and cerebellar areas in aMCI patients, particularly in those with a high level of CR. Such differences specifically concerned functional connectivity between prefrontal (BA10), parietal (BA43), and temporal (BA37) areas with various cerebellar vermian and hemispherical lobules. Altogether, these findings support the cerebellar involvement in the compensatory mechanisms related to CR.

Interestingly, starting from the widespread cerebellar connections with associative cortical areas, the role of the cerebellum in supporting cognitive processes has been well established in the last decades (Schmahmann et al., 2019; Sokolov et al., 2017), and allowed advancing the existence of cerebellar cognitive reserve (CbCR) (Mitoma et al., 2020, 2022; Arleo et al., 2024; Devita et al., 2024; Samstag et al., 2025). It basically refers to the cerebellar ability to compensate for and restore functions in response to damage (Mitoma et al., 2020; Gelfo and Petrosini, 2022; Gelfo et al., 2023). Animal models and clinical studies advanced the idea that cognitive enrichment enhances the functional efficiency of the cerebellum (Gelfo et al., 2016; Bordignon et al., 2021). Thus, building on this framework, the CbCR emerges as a specialized extension of CR, specifically attributing reserve capacity to the cerebellum’s contributions to cognitive processes. While CR traditionally emphasizes cerebral mechanisms, CbCR operationalizes the cerebellum’s role in cognitive compensation through its extensive connections with subcortical and associative cortical areas, enabling enhanced functional efficiency and recruitment during tasks (Schmahmann et al., 2019; Sokolov et al., 2017). This distinction leads to considering CbCR as not a separate construct but a cerebellar subdomain of CR. Consequently, higher CbCR indices, such as cerebellar grey matter volume or connectivity strength in regions like crus I and lobules IV–VII, correlate with overall CR proxies (e.g., education, cognitive performance, leisure activities, occupational engagement, etc.) and predict resilience to cognitive decline after neurological damage (Serra et al., 2017; Mitoma et al., 2020, 2022). Therefore, differentiating CbCR enables a more precise evaluation of how cerebellar adaptations contribute to the enhancement of the broader CR framework.

MRI techniques capable of assessing the structural connectivity between cerebellar and cortical regions are instrumental in detecting changes in connectivity. In particular, the multivariate data-driven source-based morphometry (SBM) identifies patterns of covariance of grey matter concentration (GMC) and evaluates structural networks as a whole (Xu et al., 2009; Van Assche et al., 2024; Wang et al., 2023). A recent study in MCI patients showed a reduced GMC in a widespread network, including frontal, temporal, parietal, and cerebellar regions (Setiadi et al., 2024).

To the best of our knowledge, no studies have investigated the potential effect of reserves on the GMC changes in cerebellar structural networks across the full spectrum of AD. The present research aims to examine structural connectivity changes in cerebellar-cortical networks along the AD continuum, and to evaluate the potential effect of reserves on these changes. For the present study, we considered the years of formal education as a proxy of CR. Education is a major determinant of brain resilience, associated with enhanced neocortical connectivity and sustained engagement in cognitively stimulating activities across the lifespan (Stern et al., 2020), supporting adaptive brain plasticity, increasing resistance to neurodegeneration, and also promoting efficient cognitive strategies.

Remarkably, education exerts a global enhancing effect on cognitive functioning rather than domain-specific influences, supporting broad cognitive efficiency and flexible cognitive strategies across multiple domains. This characteristic makes education particularly suitable as an indicator of CbCR, given the role of the cerebellum as a global regulator of higher-order cognitive processes (Schmahmann et al., 2019; Sokolov et al., 2017). In fact, beyond motor control, the cerebellum contributes to the modulation and optimization of cognitive functions across a wide range of domains, acting as a domain-general hub for cognitive coordination rather than supporting isolated cognitive operations.

## 2 Methods

### 2.1 Participants

A cohort of 213 participants, 59 patients with AD (M/F = 20/39; mean age = 73.3, SD = 6.6 years; mean years of formal education = 10.0, SD = 4.45), 59 patients with aMCI (M/F = 25/33; mean age = 72.3, SD = 8.20 years; mean years of formal education = 12.8, SD = 8.20), 46 individuals with SCD (M/F = 18/29; mean age = 68.2, SD = 7.27 years; mean years of formal education = 13.0, SD = 3.41), and 64 healthy elderly participants (healthy subjects: HS) (M/F = 18/31; mean age = 64.6, SD = 8.11 years; mean years of formal education = 14.3, SD = 3.88) were consecutively recruited between 2023 and 2025 from Fondazione Santa Lucia, IRCCS, Rome, Italy. To reduce the heterogeneity in the age of the participants, an age-matching procedure was carried out before statistical analyses. We obtained four groups with no significant between-group differences in the mean age distribution. A final cohort of 179 participants, 46 patients with AD (M/F = 16/30; mean age ± SD: 71.0 ± 5.4 years; mean years of formal education ± SD = 10.4 ± 4.6), 51 patients with aMCI (M/F = 20/31; age = 70.5 ± 7.3 years; education = 12.8 ± 4.1), 46 individuals with SCD (M/F = 18/28; age = 68.1 ± 7.26 years; education = 13.0 ± 3.41), and 36 healthy subjects (HS) (M/F = 17/19; age = 67.9 ± 6.7 years; education = 14.3 ± 3.6) were included in the analyses.

Except for participants with SCD and HS, AD and aMCI patients had a neurobiological diagnosis of AD based on CSF biomarkers (according to Core 1 Research criteria) (Jack et al., 2024). Clinically, aMCI patients felt into aMCI single-domain category (Albert et al., 2011), and by definition, they had not to respond to the diagnostic criteria for major cognitive disorders, while their clinical dementia rating (Hughes et al., 1982) score had not to exceed 0.5, and their Mini-Mental State Examination (MMSE) score (Folstein et al., 1975; Magni et al., 1996) had to fall within the cut-off of normality (≥23.8). SCD individuals met the following inclusion criteria according to the Jessen proposal (Jessen et al., 2014, 2020): presence of subjective memory complaint in daily living; no evidence of cognitive deficits in memory or in other cognitive domains on formal neuropsychological testing; absence of any other clinical condition accounting for their symptoms. The inclusion criteria for HS were that they had not reported any subjective cognitive complaint in daily living, they had to score normally in all cognitive domains on formal neuropsychological testing, and they had not shown any significant atrophy in their medial temporal lobes.

The recruited participants with a Hachinski score > 4 were excluded (Hachinski et al., 1975). Major systemic, psychiatric, and other neurological illnesses were also carefully investigated and excluded in all participants. Finally, participants had to be right-handed, as assessed by the Edinburgh Handedness Inventory (Büsch et al., 2010).

The study was approved by the Ethics Committee of Santa Lucia Foundation, and written informed consent was obtained from all participants and/or their legal guardians before study initiation. All procedures performed in this study are in accordance with the 1964 Helsinki declaration and its later amendments or comparable ethical standards.

### 2.2 Neuropsychological assessment

All participants underwent the Italian version of the Addenbrooke’s Cognitive Examination-Revised (ACE-R). This instrument was used to assess a range of cognitive functions, including orientation, attention, memory, fluency, language, and visuospatial abilities. Scores on the ACE-R range from 0 to 100, with a cut-off score of >66.92 indicating normal cognitive function. Additionally, the ACE-R allows for the calculation of the Mini-Mental State Examination (MMSE) score. Since the current study was focused on the cognitive reserve, neuropsychological scores were not adjusted for age and education, as previously reported (Serra et al., 2011).

### 2.3 Classification criteria to define the level of cognitive reserve

The participants were divided based on their level of formal education, which served as a proxy measure of CR. As previously described (Serra et al., 2011; Serra et al., 2017; Serra et al., 2022) within each group the years of formal education were transformed into *z*-scores, and individuals reporting a *z*-score ≤0 were considered as having a low level of CR; conversely, individuals with a *z*-score >0 were considered having a high level of CR. Following this criterion, 22 AD patients with low CR (AD-L_CR_) and 24 AD patients with high CR (AD-H_CR_), 18 aMCI patients with low CR (aMCI-L_CR_) and 33 aMCI patients with high CR (aMCI-H_CR_), 30 individuals with SCD and low CR (SCD-L_CR_) and 16 individuals with SCD and high CR (SCD-H_CR_), 19 healthy subjects with low CR (HS-L_CR_) and 17 HS with high CR (HS-H_CR_) have been categorized.

### 2.4 MRI procedure

All participants underwent a 3T MRI brain scan (MAGNETOM Prisma MRI scanner, Siemens Healthcare, Erlangen, Germany) equipped with a 64-channel head-and-neck coil. All participants underwent the following whole brain acquisitions: (a) T2-weighted turbo spin-echo (TSE) [matrix 448 × 448, FOV 220 × 220 mm^2^, 29 transversal slices, slice thickness 4 mm, repetition time (TR) = 3,490 ms, echo time (TE) = 95 ms, acquisition time 2 min 42 s]; (b) fast fluid-attenuated inversion recovery (FLAIR) [matrix 240 × 256, FOV 240 × 256 mm^2^, 176 sagittal slices, slice thickness 1 mm, TR = 8,000 ms, TE = 314 ms, inversion time (TI) = 2,350 ms, acquisition time 7 min 54 s]; (c) T1-weighted multi-echo MPRAGE sequence (MEMPRAGE) [matrix 256 × 256, FOV 256 × 256 mm^2^, 176 sagittal slices, slice thickness 1 mm, TR = 2,500 ms, TE = 1.67/3.48/5.29/7.10 ms, TI = 1,080 ms, flip angle 8°, acquisition time 7 min 27 s].

### 2.5 MRI data processing

To characterize GM differences among groups, the T1-weighted images were analysed by the multivariate data-driven source-based morphometry (SBM) approach (Xu et al., 2009). SBM extends the information provided by voxel-based morphometry (VBM) (Ashburner and Friston, 2000), integrating that derived by the independent component analysis (ICA) (McKeown and Sejnowski, 1998). By using T1-weighted images, the SBM identifies a pattern of covariance of the GMC (Gupta et al., 2019). SBM, which combines information among voxels, identifies structural networks that covary, and statistically verifies the covariance in these networks, rather than testing individual voxels separately, as VBM does (Xu et al., 2009). In practice, while univariate approaches, such as VBM, can only assess the difference in voxel groups among participants, not their mutual relationship, the multivariate approaches, such as SBM, allow for evaluating how the interrelationship among voxel groups varies across multiple groups of individuals. Therefore, while VBM provides information only on isolated brain regions, SBM provides information on groups of voxels that aggregate and form a network of brain regions. SBM leverages independent component analysis (ICA) to focus on spatially independent sources underlying the structural data (Gupta et al., 2019). In this way, it is possible to study in a multivariate fashion how GM covariance patterns change between participants.

#### 2.5.1 Data pre-processing

T1-weighted images were pre-processed using SPM12 (Wellcome Centre for Human Neuroimaging, London). The following steps were applied: (1) Segmentation of brain tissue into GM, white matter, and cerebrospinal fluid; (2) Normalization of GM images to MNI standard space; (3) Modulation of GM images to preserve local volumetric information; (4) Spatial smoothing by using an 8 mm full-width at half-maximum (FWHM) Gaussian kernel. The resulting GM images were used as input for SBM ICA analysis.

#### 2.5.2 SBM ICA analysis

SBM was applied using the ICA fMRI Toolbox (GIFT) software v4.0c^1^ to estimate 20 independent components, according to the minimum description length method (Li et al., 2007). ICA was performed by using Infomax algorithm that maximizes the statistical independence among the extracted components. More specifically, it minimizes the amount of shared information (mutual information) between them, allowing each component to represent a unique pattern of GM variation (Lee et al., 1999; Bell and Sejnowski, 1995). Extracted components were assessed using the ICASSO toolbox. We run ICASSO 20 times by using the RandInit mode. Reliability of components was quantified by using a quality index (Iq) that ranges between 0 to 1 (Himberg et al., 2004).

From the ICA decomposition, we extracted the individual-specific loadings. These loadings represent the degree to which each individual contributes to, or expresses, the spatial GM pattern identified in each independent component. Therefore, the component loadings represent the covariance among participants. Specifically, the loading values are derived from the “mixing matrix” produced by the ICA algorithm. Each row of the matrix corresponds to a participant, and each column to a component, resulting in a participant × component matrix. Higher loading values indicate a stronger expression of the associated structural pattern in a given individual. This structural pattern is expressed in terms of the grey matter concentration (GMC), a measure of GM volume. Lower or negative loadings indicate low levels of GMC, thus evidencing a superior degree of atrophy in the regions of the network. Higher or positive loadings indicate high levels of GMC, evidencing a lower degree of atrophy in the regions of the network. Specifically, negative loadings indicate less GM volumes, more atrophy, and reduced structural integrity. On the contrary, positive loadings indicate preserved GM volumes, less atrophy, and maintained structural integrity.

These loadings were used as variables of interest in statistical analyses for group comparisons and correlations. Moreover, the ICA algorithm produced a “source matrix,” which contains the distribution across voxels of the spatially independent components derived from the input GM data. Each row of this matrix corresponds to a component and represents a distinct GM structural pattern, composed of voxel-wise weights that define the spatial distribution of that pattern across the brain. These resulting spatial maps capture unique and coherent patterns of GM morphology variations, expressed to varying degrees in single individuals. The spatial maps were subsequently thresholded, reshaped into 3D images, and visualized as GM structural networks.

After ICA decomposition, we visually reviewed the 20 extracted components and selected those including cerebellar regions for the specific purpose of the study.

### 2.6 Medial temporal lobe atrophy

The Medial Temporal Lobe Atrophy scale (MTA) (Pereira et al., 2014) was employed on T1-weighted images to assess the severity of hippocampal atrophy in each participant. This scale provides a rating score from 0 to 4, with scores ≥1.5 indicating significant atrophy. For each participant, we averaged the scores obtained in the right and left hemispheres to obtain a single measure of medial-temporal lobe atrophy.

### 2.7 Statistical analyses

SPSS-25.0^2^ was used for statistical comparisons. AD, aMCI, SCD, and HS groups were compared in demographical and cognitive measures by using the one-way ANOVAs (please see Supplementary material). MANOVA has been used to assess between-group comparisons on the brain structural connectivity measures (expressed as GMC). ANOVAs on single networks and relative *post-hoc* have also been performed.

Moreover, to assess the potential effect of CR on the cognitive measures (MMSE and ACE-R scores), we used one-way ANOVAs between groups with high vs. low CR levels. In addition, to assess the potential CR effect on the cerebellar-cortical structural connectivity, we used the MANOVA model. Again, ANOVAs on single networks and relative *post-hoc* have also been performed. Finally, Pearson’s coefficients were used to determine correlations between brain structural connectivity measures, the CR measure (expressed as a continuous variable by using *z*-scores), and the measure of general cognitive efficiency (MMSE score). To account for multiple comparisons, *p*-values were adjusted using the Benjamini–Hochberg false discovery rate (FDR) procedure. An FDR threshold of *q* = 0.05 was applied.

## 3 Results

### 3.1 Demographical and clinical features

There were no significant differences among the four diagnostic groups (AD, aMCI, SCD and HS) in mean age (*F*_3,175_ = 3.36, *p* = 0.072) and in sex distribution (AD vs. aMCI: *χ*^2^ = 0.20, df = 1, *p* = 0.651; AD vs. SCD: *χ*^2^ = 0.19, df = 1, *p* = 0.665; AD vs. HS: *χ*^2^ = 1.30, df = 1, *p* = 0.254; aMCI vs. SCD: *χ*^2^ = 0.00, df = 1, *p* = 0.993; aMCI vs. HS: *χ*^2^ = 0.55, df = 1, *p* = 0.456; SCD vs. HS: *χ*^2^ = 0.54, df = 1, *p* = 0.462). As expected, there were significant differences in mean years of formal education (*F*_3,175_ = 7.19, *p* < 0.001) and MTA scores (*F*_3,175_ = 17.7, *p* < 0.001). Statistical details are reported in the Supplementary material.

Table 1 shows the demographic and clinical features of the eight groups divided according to their CR level (AD-L_CR_, AD-H_CR_, aMCI-L_CR,_ aMCI-H_CR,_ SCD-L_CR,_ SCD-H_CR,_ HS-L_CR,_ SCD-H_CR_). There were no significant differences among groups with low and high CR level in mean age (*F*_7,171_ = 2.06, *p* = 0.06) and sex distribution (all *p* ≥ 0.183), but as expected, there were significant differences in the years of formal education (*F*_7,171_ = 90.14, *p* < 0.0001) and MTA scores (*F*_7,171_ = 7.79, *p* < 0.001). All statistical details are reported in the Supplementary material.

**Table 1:** Principal demographic and clinical characteristics of the participants.

	Participants							
Mean ± SD	AD-L_CR_	AD-H_CR_	aMCI-L_CR_	aMCI-H_CR_	SCD-L_CR_	SCD-H_CR_	HS-L_CR_	HS-H_CR_
*N*	22	24	18	33	30	16	19	17
Age years	72.4 ± 4.3	69.4 ± 6.2	69.4 ± 8.8	71.1 ± 6.5	68.2 ± 6.7	67.4 ± 8.1	70.0 ± 7.0	65.6 ± 5.7
Education years	6.4 ± 1.5	14.5 ± 2.2#	8.0 ± 1.4*	15.5 ± 2.4#§	11.0 ± 2.3#*§∇	16.9 ± 0.7#*§ ∞	11.4 ± 2.5#*§∇!	17.6 ± 0.8#*§∇ ∞^
Sex M/F	7/15	9/15	7/11	15/18	11/19	7/9	8/11	9/8
MTA score	2.1 ± 1.0	2.1 ± 0.9	1.3 ± 1.2	1.8 ± 1.1#	0.7 ± 0.8#*∇	0.8 ± 0.9#*∇	1.1 ± 0.8#*	1.1 ± 0.9*#

#AD-L_CR_ vs. other groups *p*-value<0.05; *AD-H_CR_ vs. other groups *p*-value<0.05; §aMCI-L_CR_ vs. other groups *p*-value <0.05; ∇aMCI-H_CR_ vs. other groups p-value <0.05; ∞SCD-L_CR_ vs. other groups *p*-value<0.05; !SCD-H_CR_ vs. other groups *p*-value <0.05; ^HS-L_CR_ vs. other groups *p*-value<0.05.

AD-L_CR_, Alzheimer’s disease patients with low cognitive reserve; AD-H_CR_, Alzheimer’s disease patients with high cognitive reserve; aMCI-L_CR_, amnestic mild cognitive impairment patients with low cognitive reserve; aMCI-H_CR_, amnestic mild cognitive impairment patients and high cognitive reserve; HS-L_CR_, healthy subjects with low cognitive reserve; HS-H_CR_, healthy subjects with high cognitive reserve; F, female; M, male; MMSE, Mini-Mental State Examination; MTA, Medial Temporal Lobe Atrophy scale.

### 3.2 Neuropsychological assessment

As shown in Table 2 there were significant differences in all considered neuropsychological measures among the eight groups. Specifically, there were significant differences in the MMSE scores (*F*_7,171_ = 38.6, *p* < 0.0001), in ACE-R total scores (*F*_7,171_ = 62.9, *p* < 0.0001), and in the different ACE-R domains: orientation/attention (*F*_7,171_ = 24.4, *p* < 0.0001), memory (*F*_7,171_ = 56.5, *p* < 0.0001), executive functions (*F*_7,171_ = 21.7, *p* < 0.0001), language (*F*_7,171_ = 23.1, *p* < 0.0001), visuo-spatial abilities (*F*_7,171_ = 10.4, *p* < 0.0001). As expected, compared to all other groups, AD patients, independently of their CR level, showed the worst performances both in general cognition and in the single ACE-R domains. Conversely, both groups of aMCI patients showed lower scores than the SCD and HS groups in general cognition and memory. In addition, aMCI-L_CR_ patients showed significantly lower scores in the executive functions compared to the SCD and HS groups. Both groups of SCD and HS did not show significant differences between them. *Post-hoc* comparisons, as well as the results on neuropsychological measures of four diagnostic groups, are reported in the Supplementary material.

**Table 2:** Performance obtained by participants divided according their CR level at neuropsychological tests.

Scores: Mean ± SD	Participants							
	AD-L_CR_	AD-H_CR_	aMCI-L_CR_	aMCI-H_CR_	SCD-L_CR_	SCD-H_CR_	HS-L_CR_	HS-H_CR_
MMSE	23.2 ± 3.6	22.8 ± 3.6	26.5 ± 2.3#*	26.9 ± 1.8#*	29.5 ± 0.7#*§∇	29.6 ± 0.6#*§∇	29.7 ± 0.7#*§∇	29.6 ± 0.8#§*∇
ACE-R total	61.1 ± 9.7	62.2 ± 12.0	79.7 ± 10.0#*	83.4 ± 9.2#*	92.2 ± 5.3#*§∇	92.7 ± 4.8#*§∇	93.3 ± 5.0#*§∇	95.5 ± 3.3#*§∇
Orientation/Attention	13.8 ± 2.9	14.6 ± 2.2	16.7 ± 1.6#*	16.7 ± 1.4#*	17.9 ± 0.1#*	17.5 ± 0.2#*	17.9 ± 0.2#*	17.8 ± 0.3#*
Memory	9.2 ± 4.6	9.3 ± 3.6	16.1 ± 5.5#*	17.7 ± 4.8#*	22.9 ± 3.0#*§∇	23.0 ± 2.6#*§∇	23.4 ± 3.2#*§∇	24.6 ± 1.4#*§∇
Executive functions	6.0 ± 2.1	6.0 ± 3.0	8.4 ± 3.1#*	10.2 ± 2.4#*	10.7 ± 2.3#*§	11.3 ± 1.8#*§	11.5 ± 1.7#*§	11.8 ± 1.6#*§
Language	21.3 ± 3.2	19.8 ± 4.6	24.9 ± 1.4#*	24.5 ± 1.7#*	25.6 ± 0.9#*	25.7 ± 0.6#*	25.6 ± 0.7#*	25.9 ± 0.2#*
Visuo-spatial abilities	10.4 ± 2.4	12.5 ± 3.8	14.2 ± 3.7#	14.1 ± 2.1#	15.0 ± 1.7#*	15.2 ± 1.1#*	14.8 ± 1.5#*	15.3 ± 1.2#*

#AD-L_CR_ vs. other groups *p*-value <0.05; *AD-H_CR_ vs. other groups *p*-value <0.05; §aMCI-L_CR_ vs. other groups *p*-value <0.05; ∇aMCI-H_CR_ vs. other groups *p*-value <0.05; ∞SCD-L_CR_ vs. other groups *p*-value <0.05; !SCD-H_CR_ vs. other groups *p*-value <0.05; ^HS-L_CR_ vs. other groups *p*-value <0.05.

MMSE, Mini Mental State Examination; ACE-R, Addenbrooke’s Cognitive Examination-Revised; AD-L_CR_, Alzheimer’s disease patients with low cognitive reserve; AD-H_CR_, Alzheimer’s disease patients with high cognitive reserve; aMCI-L_CR_, amnestic mild cognitive impairment patients with low cognitive reserve; aMCI-H_CR_, amnestic mild cognitive impairment patients and high cognitive reserve; HS-L_CR_, healthy subjects with low cognitive reserve; HS-H_CR_, healthy subjects with high cognitive reserve.

### 3.3 Source-based morphometry

As summarized in the Supplementary Figure S1, all ICA components showed good convergence and stable decomposition. In particular, all components, including those selected in this study (highlighted with a red circle in the figure), showed an Iq >0.95, indicating a high reliability of the brain structural networks extracted.

**Figure 1:**
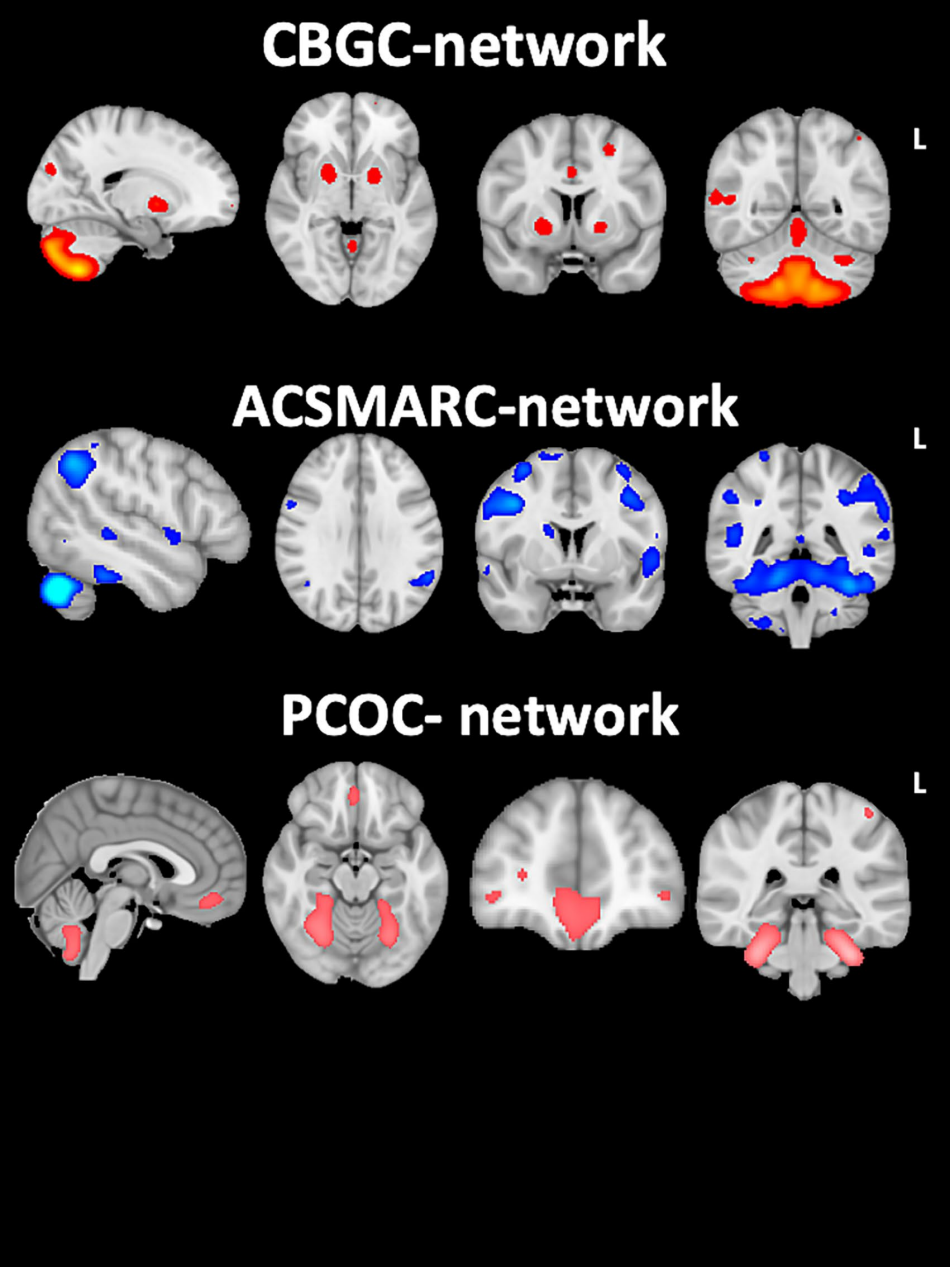
Cerebellar-cortical networks. SBM analysis identified three main components of grey matter covariance (GMC) involving cerebellar-cortical regions. The cerebellum-basal ganglia-cingulum (CBGC) network is shown above, involving: anterior cerebellar lobe (lobules I–IV), posterior cerebellar lobe (lobules VI–VIII), basal ganglia, and cingulum. The anterior cerebellum-supplementary motor area-retrosplenial cortex (ACSMARC) network is shown in the middle: cerebellar lobule V and crus I, supplementary motor area, and retrosplenial cortex. The posterior cerebellum-orbitofrontal cortex (PCOC) network is shown at the bottom: cerebellar lobules I–IV and mainly IX, and orbitofrontal cortex. This figure illustrates the spatial component maps describing the topography of structural covariation of grey matter concentration into the three cerebellar-cortical networks. The maps do not include any quantitative information and are reported only for illustrative purpose. They are overlaid onto MNI-152 template by using FMRIB software library-FSL (https://fsl.fmrib.ox.ac.uk/fsl/). CBGC, cerebellum-basal ganglia-cingulum network; ACSMARC, anterior cerebellum-supplementary motor area-retrosplenial cortex network; PCOC, posterior cerebellum-orbitofrontal cortex network.

As shown in Figure 1, SBM analysis identified three main components of GMC involving cerebellar-cortical regions. The first component included the GMC of the following regions: cerebellar anterior lobe (lobules I–IV), cerebellar posterior lobe (lobules VI–VIII), basal ganglia, and cingulum. We named this component the cerebellum-basal ganglia-cingulum (CBGC) network. The second component included the GMC of the cerebellar lobule V and crus I, supplementary motor area, and retrosplenial cortex. We named this component the anterior cerebellum-supplementary motor area-retrosplenial cortex (ACSMARC) network. Finally, the third component included the GMC of lobules I–IV and mainly IX and the orbitofrontal cortex. To emphasize the different cerebellar components with respect to the previously described networks, we named this circuit the posterior cerebellum-orbitofrontal cortex (PCOC) network.

**Figure 2:**
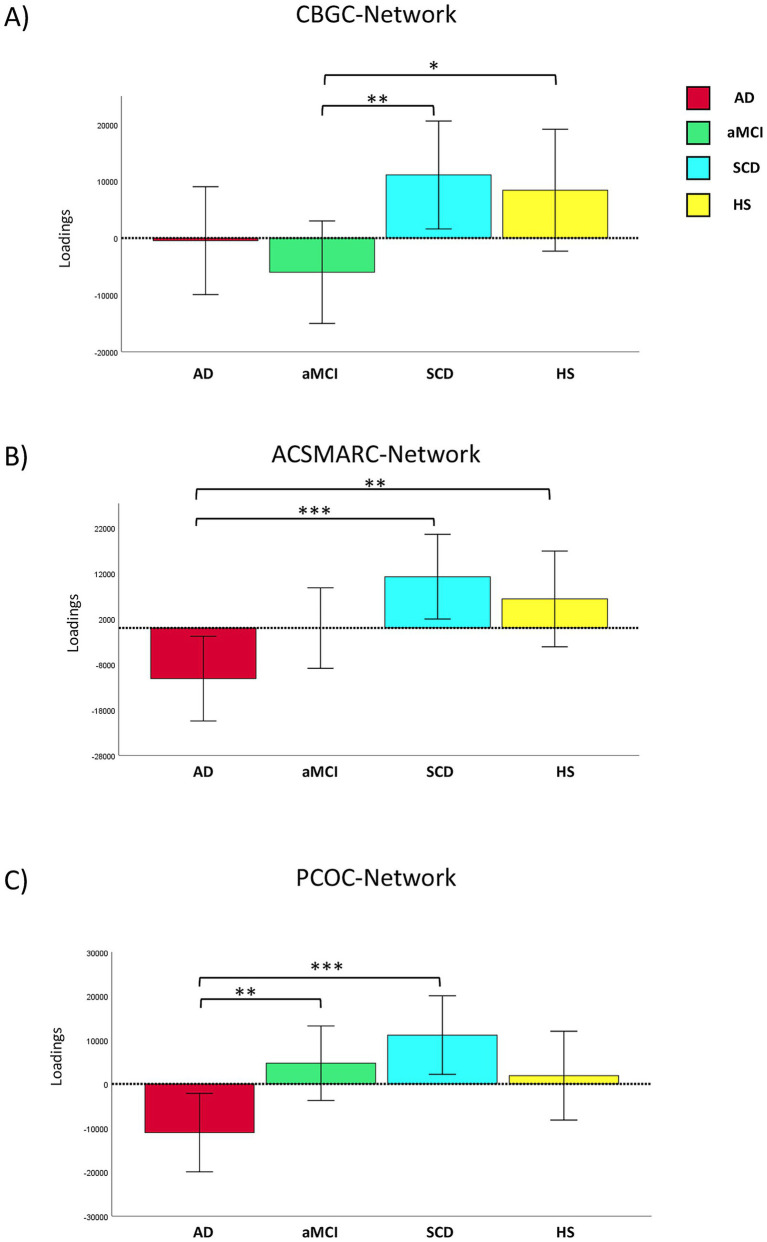
Pattern of grey matter covariance in cerebellar-cortical networks. Group differences obtained by ANOVAs, in grey matter covariance (GMC) across the three cerebellar–cortical networks in the four study groups: AD (red), aMCI (green), SCD (light blue), and HS (yellow). Significant effects (^*^p-level ≤0.05; ^**^p-level ≤0.01; ^***^p-level ≤0.001) were found in all three networks (CBGC, ACSMARC, and PCOC). Patients with aMCI showed reduced GMC covariance in the CBGC network (panel **A**) compared to the SCD and HS groups. AD and aMCI patients showed reduced GMC covariance in ACSMARC (panel **B**) and PCOC (panel **C**) networks compared to SCD and HS. No differences emerged between SCD and HS groups. See text for further details. AD, Alzheimer’s disease patients; aMCI, amnestic mild cognitive impairment patients; SCD, subjective cognitive decline individuals; HS, healthy subjects; CBGC, cerebellum-basal ganglia-cingulum network; ACSMARC, anterior cerebellum-supplementary motor area-retrosplenial cortex network; PCOC, posterior cerebellum-orbitofrontal cortex network.

MANOVA on GMC of the three cerebellar cortical networks showed a significant group effect (Wilks’ *λ* = 0.866, *F*_9,421_ = 2.84, *p* = 0.003, *η*^2^*p* = 0.047). ANOVAs showed significant group effects in the covariation of GMC in the CBGC (*F*_3,175_ = 2.82, *p* = 0.041, *η*^2^*p* = 0.046), the ACSMARC (*F*_3,175_ = 4.24, *p* = 0.006, *η*^2^*p* = 0.068), and the PCOC (*F*_3,175_ = 4.38, *p* = 0.005, *η*^2^*p* = 0.070) networks. *Post-hoc* analyses showed in CBGC-network a significant reduction of GMC in aMCI patients compared to SCD (*p* = 0.010; CI 95%: −30063.6; −4201.9) and HS (*p* = 0.041; CI 95%: −28291.0; −605.1) groups; in ACSMARC-network significant GMC reduction was found in AD patients compared to SCD (*p* = 0.001; CI 95%: −35415.8; −9411.4) and HS (*p* = 0.014; CI 95%: −31401.5; −3649.9) groups; finally, in PCOC-network significant GMC reduction was found in AD patients compared to aMCI (*p* = 0.011; CI 95%: −27911.4; −3618.9) and SCD (*p* = 0.001; CI 95%: −34625.5; −9714.8). Notably, no significant differences were observed between SCD and HS groups in any network (Figure 2).

**Figure 3:**
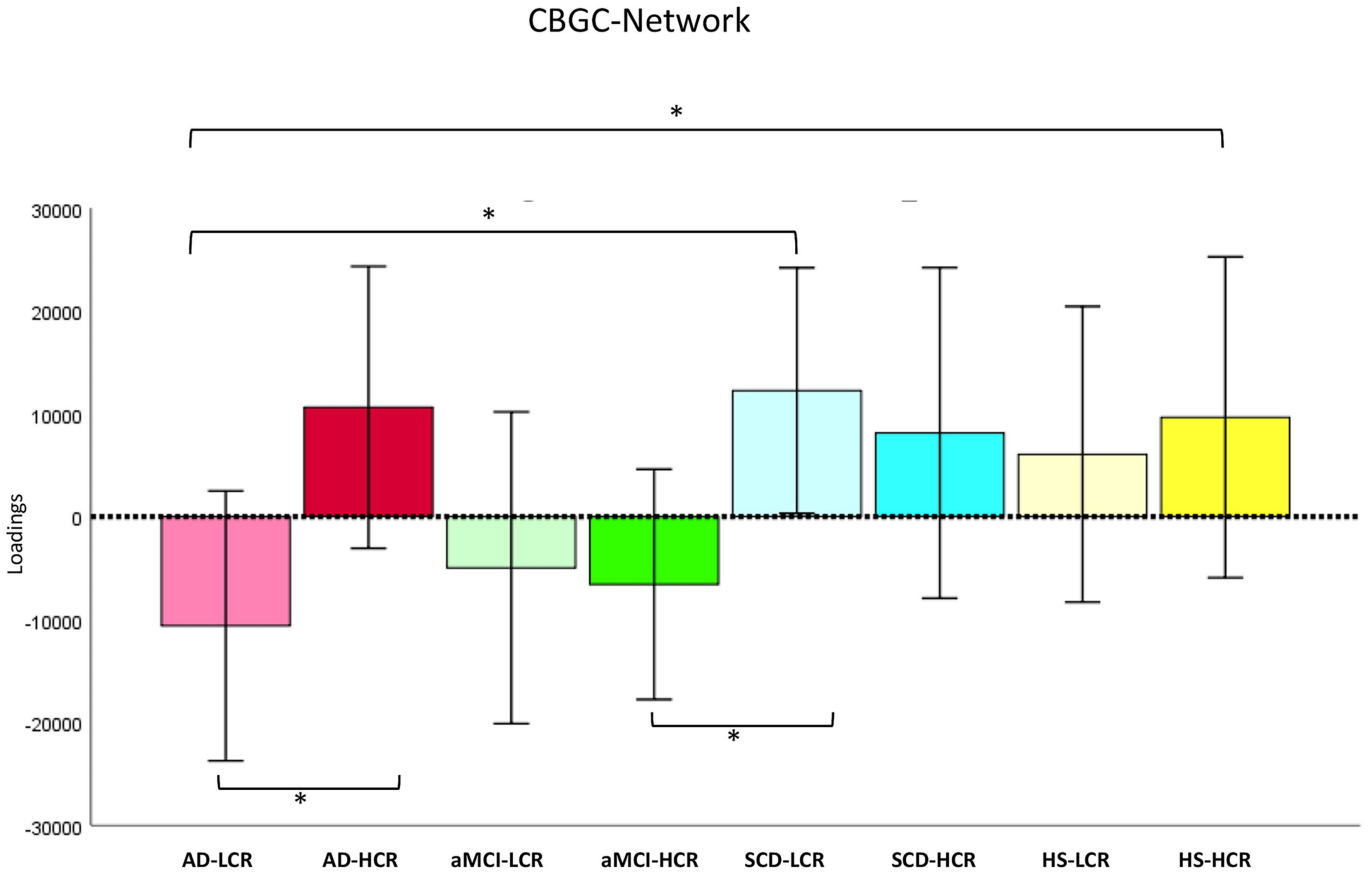
Cognitive reserve modulates grey matter covariance in the CBGC network. The figure illustrates the *post-hoc* results obtained by ANOVA to assess the effect of cognitive reserve (CR) on grey matter covariance (GMC) in the CBGC network. Significant group effects (^*^p-level ≤0.05) were found due to the reduced GMC observed in AD-L_CR_ compared to AD-H_CR_ patients, SCD-L_CR_, and HS-H_CR_. Additionally, aMCI-H_CR_ patients showed reduced GMC compared to SCD-L_CR_. See text for further details. AD-H_CR_, Alzheimer’s disease patients with high cognitive reserve; AD-L_CR_, Alzheimer’s disease patients with low cognitive reserve; aMCI-H_CR_, amnestic-mild cognitive impairment patients with high cognitive reserve; aMCI-L_CR_, amnestic-mild cognitive impairment patients with low cognitive reserve; SCD-H_CR_, subjective cognitive decline individuals with high cognitive reserve; SCD-L_CR_, subjective cognitive decline individuals with low cognitive reserve; HS-H_CR_, healthy subjects with high cognitive reserve; HS-L_CR_, healthy subjects with low cognitive reserve; CBGC, cerebellum-basal ganglia-cingulum network.

**Figure 4:**
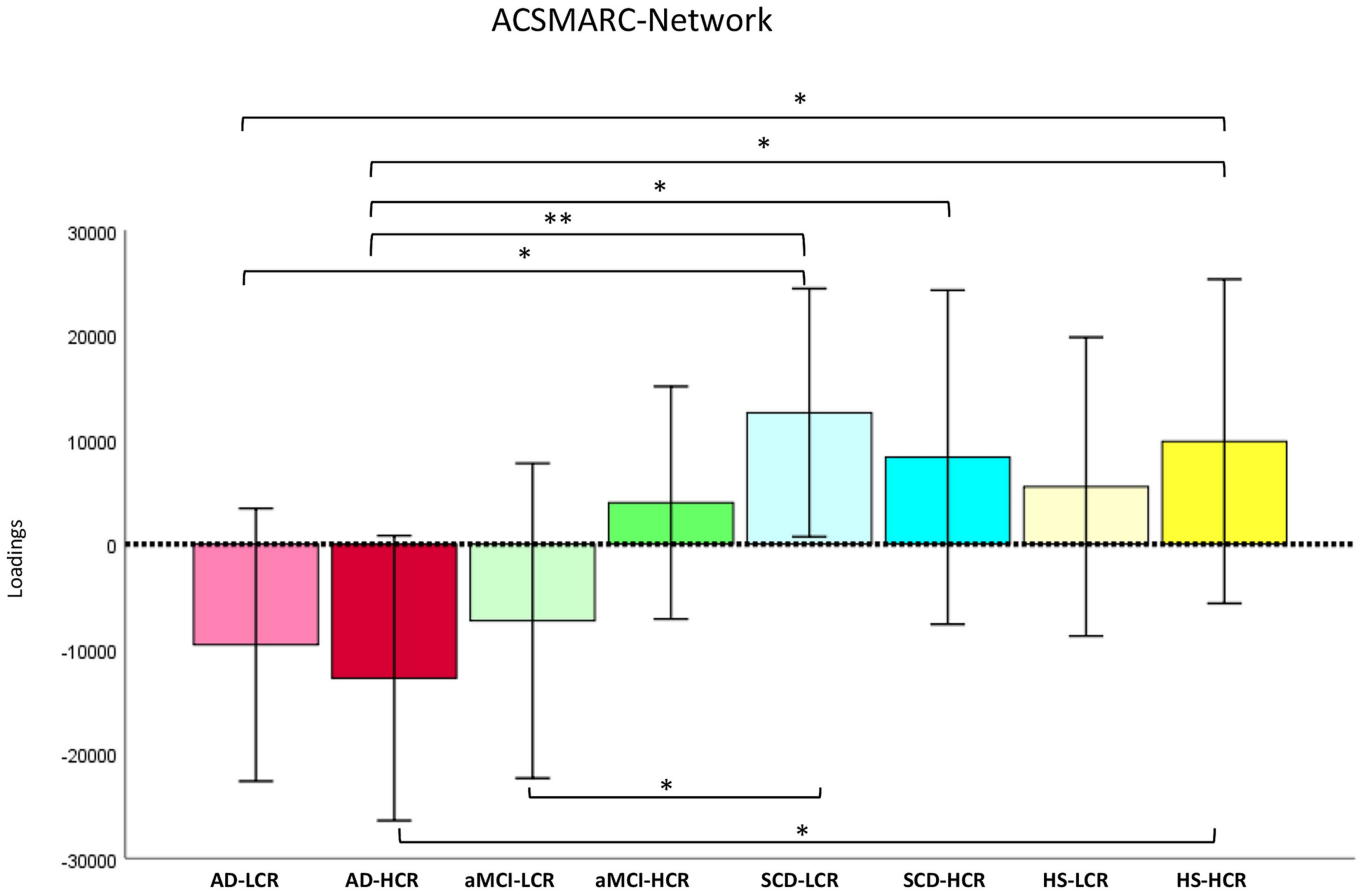
Cognitive reserve modulates grey matter covariance in the ACSMARC network. The figure illustrates the *post-hoc* results obtained by ANOVA to assess the effect of cognitive reserve (CR) on grey matter covariance (GMC) in the ACSMARC network. Significant group effects (^*^p-level ≤0.05; ^**^p-level ≤0.01) were found due to the reduction of GMC observed in the AD-L_CR_ group compared to SCD-L_CR_ and HS-H_CR_ groups, while the AD-H_CR_ group showed reduced GMC compared to SCD-LCR, SCD-HCR, and HS-HCR groups, respectively. Conversely, SCD-L_CR_ individuals displayed increased GMC relative to aMCI-L_CR_ patients. See text for further details. AD-H_CR_, Alzheimer’s disease patients with high cognitive reserve; AD-L_CR_, Alzheimer’s disease patients with low cognitive reserve; aMCI-H_CR_, amnestic-mild cognitive impairment patients with high cognitive reserve; aMCI-L_CR_, amnestic-mild cognitive impairment patients with low cognitive reserve; SCD-H_CR_, subjective cognitive decline individuals with high cognitive reserve; SCD-L_CR_, subjective cognitive decline individuals with low cognitive reserve; HS-H_CR_, healthy subjects with high cognitive reserve; HS-L_CR_, healthy subjects with low cognitive reserve; ACSMARC, anterior cerebellum-supplementary motor area-retrosplenial cortex network.

Remarkably, when considering the CR effect on the GMC in the three different networks MANOVA revealed significant group effects (Wilks’ *λ* = 0.812, *F*_21,485_ = 1.74, *p* = 0.022, *η*^2^*p* = 0.067). ANOVAs showed significant group effects in the covariation of GMC in the CBGC (*F*_7,171_ = 2.06, *p* = 0.05, *η*^2^*p* = 0.078) network (Figure 3). *Post-hoc* showed significant GMC reduction in AD-L_CR_ patients compared to AD-H_CR_ (*p* = 0.026; CI 95%: −39970.4; −2517.8) compared to SCD-L_CR_ (*p* = 0.011; CI 95%: −40383.0; −5368.1) and compared to HS-H_CR_ (*p* = 0.048; CI 95%: −40387.6; −163.8), in aMCI-H_CR_ compared to SCD-L_CR_ (*p* = 0.022; CI 95%: −34989.6; −2692.7) (see Figure 3). ANOVAs showed significant group effects in the covariation of GMC in the ACSMARC (*F*_7,171_ = 2.08, *p* = 0.048, *η*^2^*p* = 0.079) network (Figure 4). *Post-hoc* showed significant GMC reduction in AD-L_CR_ patients compared to SCD-L_CR_ (*p* = 0.013; CI 95%: −39573.4; −4798.3) and to HS-H_CR_ (*p* = 0.05; CI 95%: −39417.6; −530.6), in AD-H_CR_ patients compared to SCD-L_CR_ (*p* = 0.006; CI 95%: −43179.3; −7549.7) and in comparison to SCD-H_CR_ (*p* = 0.046; CI 95%: −41803.6; −398.3) and compared to HS-H_CR_ (*p* = 0.030; CI 95%: −42969.2; −2275.1); SCD-L_CR_ individual showed significant GMC increase in comparison aMCI-L_CR_ (*p* = 0.039; CI 95%: 982.4; 38796.1) (see Figure 4).

**Figure 5:**
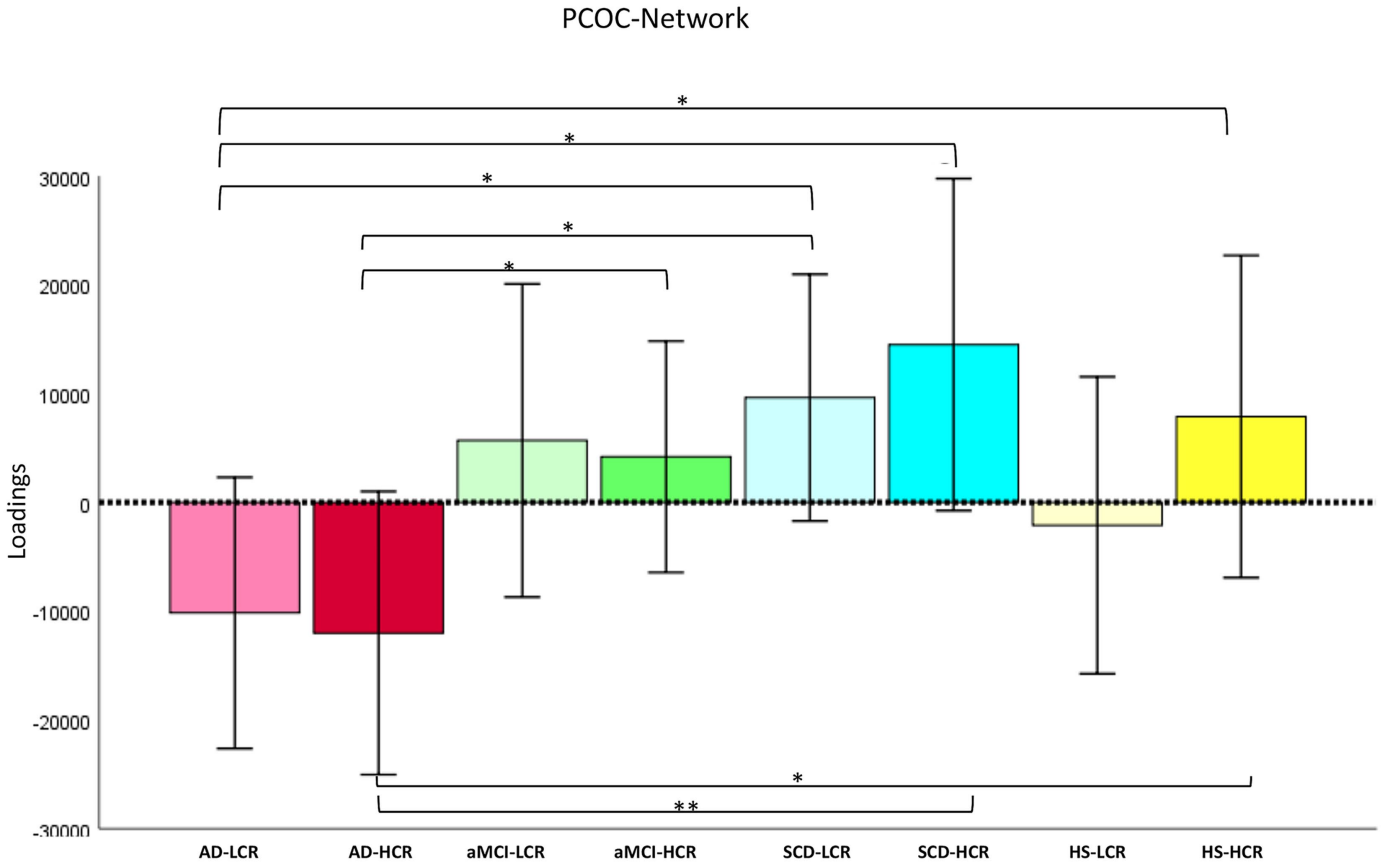
Cognitive reserve modulates grey matter covariance in the PCOC network. The figure illustrates the *post-hoc* results obtained by ANOVA to assess the effect of cognitive reserve (CR) on grey matter covariance (GMC) in the PCOC network. Significant group effects (^*^p-level ≤0.05; ^**^p-level ≤0.01) were found because AD-L_CR_ patients exhibited lower GMC compared to SCD-L_CR_, SCD-H_CR_, and HS-H_CR_ groups. Moreover, the AD-H_CR_ group showed reduced GMC compared to aMCI-H_CR_, SCD-L_CR_, SCD-H_CR_, and HS-H_CR_. See text for further details. AD-H_CR_, Alzheimer’s disease patients with high cognitive reserve; AD-L_CR_, Alzheimer’s disease patients with low cognitive reserve; aMCI-H_CR_, amnestic-mild cognitive impairment patients with high cognitive reserve; aMCI-L_CR_, amnestic-mild cognitive impairment patients with low cognitive reserve; SCD-H_CR_, subjective cognitive decline individuals with high cognitive reserve; SCD-L_CR_, subjective cognitive decline individuals with low cognitive reserve; HS-H_CR_, healthy subjects with high cognitive reserve; HS-L_CR_, healthy subjects with low cognitive reserve; PCOC, posterior cerebellum-orbitofrontal cortex network.

Finally, ANOVAs showed significant group effects in the covariation of GMC in the PCOC (*F*_7,171_ = 2.08, *p* = 0.048 *η*^2^*p* = 0.079) network (Figure 5). *Post-hoc* showed significant GMC reduction in AD-L_CR_ patients compared to SCD-L_CR_ (*p* = 0.020; CI 95%: −36426.7; −3162.4) and to SCD-H_CR_ (*p* = 0.013; CI 95%: −44117.1; −5211.8) and to HS-H_CR_ (*p* = 0.050; CI 95%: −530.6; −4201.9), AD-H_CR_ patients compared to aMCI-H_CR_ (*p* = 0.05; CI 95%: −32792.9; −385.6), to SCD-L_CR_ (*p* = 0.013; CI 95%: −38689.6; −4608.1) and to SCD-H_CR_ (*p* = 0.009; CI 95%: −46321.9; −6715.5) and to HS-H_CR_ (*p* = 0.045; CI 95%: −39371.6; −445.5) (see Figure 5).

#### 3.3.1 Correlations

After FDR correction, significant results were restricted to tests with adjusted *p*-values <0.05, including raw values with *p*-values from ≤0.00001 to ≤0.01; all other comparisons did not survive FDR corrections. As shown in Table 3, when analysing the entire sample (panel A), we found significant positive correlations between the MMSE scores and the GMC pattern across the three networks. There were also positive correlations among the GMC of the three networks. Furthermore, in the AD group (panel B), GMC of the ACSMARC network displayed positive correlations with the GMC of CBGC and PCOC networks. Moreover, the GMC of the CBGC network correlated positively with CR *z*-scores. Positive correlations between the GMC of the three networks were observed in the aMCI (panel C) and HS (panel E) groups. No significant correlation was found in the SCD individuals (panel D).

**Table 3:** Correlations between cerebellar-cortical structural connectivity, cognitive measures and CR levels.

(A) All groups	MMSE	CBGC network	ACSMARC network	PCOC network
CBGC-network	***r* = 0.223** ***p* = 0.001** ***p***_**FDR**_ **= 0.007**	—	—	—
ACSMARC-network	***r* = 0.328** ***p* < 0.001** ***p***_**FDR**_ **< 0.0001**	*r* = 0.416 ***p* < 0.001** ***p***_**FDR**_ **< 0.0001**	—	—
PCOC-network	***r* = 0.271** ***p* < 0.001** ***p***_**FDR**_ **< 0.0001**	*r* = 0.341 ***p* < 0.001** ***p***_**FDR**_ **< 0.0001**	*r* = 0.453 ***p* < 0.001** ***p***_**FDR**_ **< 0.0001**	—
CR *z*-score	*r* = 0.111 *p* = 0.156	*r* = 0.168 *p* = 0.025	*r* = 0.067 *p* = 0.372	*r* = 0.129 *p* = 0.084

(B) AD patients	MMSE	CBGC network	ACSMARC network	PCOC network
CBGC-network	*r* = 0.143 *p* = 0.372	—	—	—
ACSMARC-network	*r* = 0.297 *p* = 0.059	*r* = 0.376 *p* = 0.010 ***p***_**FDR**_ **= 0.039**	—	—
PCOC-network	*r* = 0.27 *p* = 0.866	*r* = 0.326 *p* = 0.027	*r* = 0.376 *p* = 0.010 ***p***_**FDR**_ **= 0.039**	—
CR *z*-score	*r* = 0.202 *p* = 0.205	***r* = 0.379** ***p* = 0.009** ***p***_**FDR**_ **= 0.038**	*r* = 0.114 *p* = 0.449	*r* = 0.123 *p* = 0.415

(C) aMCI patients	MMSE	CBGC network	ACSMARC network	PCOC network
CBGC-network	*r* = 0.216 *p* = 0.155	—	—	
ACSMARC-network	*r* = 0.242 *p* = 0.109	*r* = 0.469 *p* = 0.001 ***p***_**FDR**_ **= 0.007**	—	—
PCOC-network	*r* = 0.196 *p* = 0.197	*r* = 0.340 *p* = 0.010 ***p***_**FDR**_ **= 0.039**	*r* = 0.685 *p* < 0.001 ***p***_**FDR**_ **< 0.0001**	—
CR *z*-score	*r* = 0.229 *p* = 0.131	*r* = 0.069 *p* = 0.633	*r* = 0.148 *p* = 0.301	*r* = −0.019 *p* = 0.896

(D) SCD individuals	MMSE	CBGC network	ACSMARC network	PCOC network
CBGC-network	*r* = 0.216 *p* = 0.169	—	—	—
ACSMARC-network	*r* = −0.03 *p* = 0.852	*r* = 0.32 *p* = 0.029	—	—
PCOC-network	*r* = 0.24 *p* = 0.124	*r* = 0.24 *p* = 0.108	*r* = 0.23 *p* = 0.120	—
CR *z*-score	*r* = 0.04 *p* = 0.795	*r* = 0.05 *p* = 0.726	*r* = −0.01 *p* = 0.931	*r* = 0.29 *p* = 0.053

(E) HS	MMSE	CBGC network	ACSMARC network	PCOC network
CBGC-network	*r* = 0.190 *p* = 0.274	—	—	—
ACSMARC-network	*r* = 0.142 *p* = 0.415	*r* = 0.487 *p* = 0.003 ***p***_**FDR**_ **= 0.017**	—	—
PCOC-network	*r* = 0.208 *p* = 0.231	*r* = 0.492 *p* = 0.002 ***p***_**FDR**_ **= 0.012**	*r* = 0.446 *p* = 0.006 ***p***_**FDR**_ **= 0.027**	—
CR *z*-score	*r* = 0.301 *p* = 0.079	*r* = 0.109 *p* = 0.527	*r* = 0.067 *p* = 0.699	*r* = 0.161 *p* = 0.347

CR, cognitive reserve; MMSE, Mini Mental State Examination; AD, Alzheimer’s disease; aMCI, amnestic mild cognitive impairment; HS, healthy subjects; SCD, subjective cognitive decline; CBGC, cerebellum-basal ganglia-cingulum; ACSMARC, anterior cerebellum-supplementary motor area-retrosplenial cortex; PCOC, posterior cerebellum-orbitofrotal cortex. Bold values indicate significant results that survive multiple comparisons.

## 4 Discussion

The present study identified significant changes in the structural connectivity of cerebellar-cortical networks along the AD continuum. Our results confirmed the expected cognitive differences among groups, with AD patients showing the worst performances across all domains (independently of their CR levels), followed by aMCI patients in general cognition and memory (again independently of their CR levels). This lack of significant differences indicates that our patients did not improve their cognitive functioning by putting into action compensatory mechanisms or an efficient use of neural resources. However, we cannot neglect that the absence of CR effects on cognition in the patients could reflect a comparable degree of disease severity at the clinical stage examined. Not surprisingly, aMCI-L_CR_ patients showed lower performance than the SCD and HS groups in the executive functions. Indeed, frontal functions are known to be particularly sensitive to the presence of neurodegeneration since the early stage (Dos Santos et al., 2020). Moreover, SCD participants, as well as HS, did not exhibit any CR effect, suggesting that when objective cognitive impairment has not yet become apparent, the differences in global or domain-specific cognitive performances are not yet measurable, suggesting that the CR compensatory effects are still weakly engaged. Thus, it is possible that the use of more sensitive and refined neuropsychological assessments could allow for the detection of the potential impact of cognitive reserve. Finally, the lack of differences in cognitive functioning in the two groups of HS is supported by the hypothesized maintenance of cognitive efficiency due to the integrity of neural mechanisms (Stern et al., 2020).

### 4.1 Cerebellar-cortical network architecture

By SBM analysis, we identified three distinct structural cerebellar-cortical networks showing a stepwise reduction of GMC covariance along the cognitive decline spectrum, a prerequisite to investigating the potential CbCR role in modulating cognitive functions in AD stages.

#### 4.1.1 CBGC network

This network exhibited reduced connectivity in aMCI patients compared to SCD and HS groups. The widespread GM reduction involving the cerebellum and cortical areas along the AD continuum aligns with previous VBM studies (Toniolo et al., 2018; Colloby et al., 2014) showing reduced volumes of similar vermian and paravermian regions. Note that the CBGC network is involved in high-order cognitive functions, given that the basal ganglia and cingulum are brain areas involved in several cognitive functions, including executive functions (Brown et al., 1997) and memory (Zuo et al., 2025). Furthermore, cerebellar vermis, mainly in its posterior regions, has been recognized to be involved in several non-motor functions, including emotional regulation (Pierce et al., 2023), sustained attention (Townsend et al., 1999), social cognition (Van Overwalle et al., 2020), and language processing (Turker et al., 2023), supporting thus its inclusion within the framework of the cerebellar cognitive affective syndrome (CCAS) (Schmahmann, 2004) and reinforcing the cerebellar role beyond motor coordination toward an integrated model of cognitive-emotional regulation. The absence of difference between SCD and HS groups suggests that this network may remain structurally intact in the early, subjective phases of decline, becoming disrupted only with the onset of objective cognitive impairment, as occurring in the aMCI stage. As a final note, we did not find any difference in the CBGC network in the AD group. A “floor effect” (Figure 2) may be due to a GMC non-consensual covariation into the network regions due to several factors, not least a possible reserve effect, or non-uniform regional alterations among patients, or (theoretically but less plausibly) no involvement of these brain regions in AD pathology.

#### 4.1.2 ACSMARC network

This network exhibited decreased connectivity in AD patients compared to all groups. Even if lobule V has traditionally been associated with motor coordination (Chao et al., 2021), its contribution to temporal sequencing and attentional control suggests its potential role in cognitively demanding motor tasks (Stoodley et al., 2012). In contrast, since crus I is a supramodal area involved in executive functioning and language, its disruption may result in higher-order deficits typical of AD. The supplementary motor area, beyond the motor functions, is implicated in motor planning, response inhibition, and cognitive control of action, linking it to goal-directed behavior. Finally, the retrosplenial cortex, a key component of the default mode network, plays a critical role in spatial orientation and episodic memory retrieval, functions not by chance, representing the earliest cognitive domains affected in AD. As a further note, the lack of difference in the ACSMARC network in the aMCI group may be interpreted as reported above (Figure 2). Briefly, the observed gradient of alterations of the ACSMARC network indicates its heightened sensitivity to early pathological changes, a vulnerability that reflects in the disruption of integrated motor-cognitive-memory functions.

#### 4.1.3 PCOC network

This network showed significant differences in AD patients in comparison to aMCI and SCD groups. The regions included in the PCOC network are known to be crucial for cognition. Although cerebellar lobules I–IV are classically linked to sensorimotor control (Schmahmann, 1996; Kipping et al., 2013), their hypothesized role (Sokolov et al., 2017) in sustaining attention during movement may support their involvement in automatic motor-cognitive processes, making them vulnerable to AD impairment. Moreover, lobule IX participates in visuospatial integration (Stephen et al., 2018; Ramanoel et al., 2018) and in the default mode network (Stephen et al., 2018; Habas et al., 2009), suggesting its role in self-referential cognition, spatial orientation, and episodic memory. Finally, the orbitofrontal cortex is crucially involved in reward-based decision making, emotional regulation, and behavioral flexibility, all functions impaired in AD patients, emphasizing its integrative role at the crossroads of emotion, motivation, and cognition.

In conclusion, supported by memory dysfunctions associated with functional connectivity changes between the dentate nucleus and temporal regions previously described (Olivito et al., 2020), we hypothesize that, in addition to functional abnormalities, changes in structural connectivity of cerebellar-cortical networks can also occur in AD pathology. Interestingly, the cerebellar-cortical connections are known to be particularly vulnerable to the aging process as demonstrated by an anatomopathological study, showing a selective aging-related loss of Purkinje and granule cells resulting in significant atrophy of the anterior cerebellar lobe (Andersen et al., 2003; Yu et al., 2017).

### 4.2 Effects of cerebellar cognitive reserve on structural connectivity

The CR significantly influenced structural connectivity in different cerebellar-cortical circuits in both groups of AD and aMCI patients. In particular, we observed an opposite effect of CR in AD patients on the GMC of the CBGC (Figure 3) network and in aMCI patients on the GMC of the ACSMARC network (Figure 4), respectively. Interestingly, just these groups showed a “floor effect” in the GMC of the same networks when CR levels were not considered (Figure 2). The different GMCs in low and high CR individuals indicated that patients with LCR showed negative loadings that may denote a homogeneous atrophy of all network regions. Conversely, in patients with HCR, the positive loadings might denote that all network regions are unaffected (less plausible in the case of AD pathology). Alternatively, positive loadings may denote the presence of an “inhomogeneous pattern” featured by some network regions already affected by significant atrophy (as expected in the presence of AD neurodegeneration), together with other regions still unaffected. Thus, the present results may be linked to a modulatory effect of the reserve exerted in specific networks (CBGC and ACSMARC) and clinical stages (AD and aMCI). In other words, the differences observed among AD-L_CR_ and AD-H_CR_ compared to the other groups, as well as the differences observed among aMCI-L_CR_ and aMCI-H_CR_ compared to SCD and HS groups, seem to be due to the clinical diagnosis rather than a CR effect. Conversely, the differences observed within the same groups with different CR levels appear to be linked to the modulatory effect of the reserve itself, rather than the clinical diagnosis. This effect is particularly evident in the AD groups and (even if more slightly) in the aMCI groups. Ultimately, individuals with low CR showed greater network vulnerability, leading to a faster network degradation; on the contrary, individuals with high CR showed more resilience in the structure of the network, also in the presence of neuropathology. This interpretation is supported by the lack of significant differences in all networks analysed in SCD and HS groups with Low and High CR, highlighting that these networks began to be disrupted as the disease progresses. Moreover, CR is known to modulate functional reorganization more readily than structural architecture, particularly when pathology is subtle or absent. As a result, reserve-related effects may manifest predominantly at the functional level, while the structure of the networks remains relatively homogeneous across individuals. These factors likely contribute to the absence of detectable differences in structural connectivity between high and low CR groups in the early-stage pathology (as in SCD individuals) or during healthy aging. A previous work on resting-state functional connectivity showed that the cerebellum (unfortunately without any further parcelization) exhibits a compensatory pattern characterized by functional connectivity increased in MCI patients and decreased in AD patients (Castellazzi et al., 2014). Our findings partially overlapped with those reported in this study. In fact, even in the present study, the AD patients (with both low and high CR) had clearly negative loadings in ACSMARC and PCOC networks, and aMCI patients (with both low and high CR) had positive loadings in the PCOC network. Although both structural and functional connectivity assess the network integrity, they do not capture exactly the same phenomenon; the present findings are substantially in line with Castellazzi’s results. Conversely, in the CBGC network for AD patients and in the ACSMARC network for aMCI patients, we observed an opposite pattern between participants with low and high CR as described above. As a whole, the findings of the present study suggest that differences in the structural organization of the single cerebellar networks emerge in relation to CR levels. Thus, given that the only common structure in the three networks analysed was the cerebellum, we hypothesize that the difference in the GMC of the three networks may be attributed to the cerebellar capacity to express reserve mechanisms already proposed in the recent literature (Mitoma et al., 2020, 2022; Bernard, 2022; Sadeghihassanabadi et al., 2022; Arleo et al., 2024; Devita et al., 2024; Samstag et al., 2025).

### 4.3 Network correlations and clinical implications

Significant inter-network correlations were observed in AD, aMCI, and HS, but not in the SCD individuals, who showed an association only between the GMC of CBGC and ACSMARC networks. The mutual correlation among the three networks indicates, in AD and aMCI patients, a consensual alteration of connectivity in the brain regions involved in cognitive functioning, while in HS, it indicates the full integrity of these networks, resulting in complete cognitive efficiency. Conversely, the partial inter-network correlation found in the SCD group suggests that the initial loss of collaborative functioning of the three networks no longer ensured optimal cognitive efficiency. We hypothesize that the absence of correlation between the three cerebellar-cortical networks (specifically, no correlation with the PCOC network was observed) described here could result in a subjective low cognitive self-efficacy. Therefore, one would expect the reserve to have a powerful effect in buffering the influence of the lack of cooperation between the three circuits. On the contrary, no CR effect was observed in the SCD group, leading to an apparent paradox. We speculate that this result may reflect several mechanisms, acting together or separately. Firstly, in accordance with previous studies on metacognition in SCD individuals (Jenkins et al., 2021; Chapman et al., 2022), we may suggest that they are characterized by heightened cognitive awareness and relatively preserved cognitive abilities, a “worried well” phenomenon could occur, creating a ceiling effect that may obscure CR-related differences. Secondly, the cognitive concerns of SCD individuals might trigger a compensatory hypervigilance during testing. Such a heightened attentional state could equalize their performances across CR levels. Finally, the absence of CR effects in the SCD group could be related to anxiety-related cognitive interference that could mask the underlying CR differences. In other words, the performance anxiety could impose an additional cognitive load that similarly affects all SCD participants, regardless of their CR level. The resulting noise in cognitive performance might obscure the subtle effects of CR that would otherwise be detectable in more relaxed testing conditions.

We propose that a necessary condition for cognitive efficiency is the mutual collaboration of the three cerebellar-cortical networks as occurring in HS. Obviously, when the three networks are indeed mutually collaborating but at a reduced level, as in AD and aMCI patients, the cognitive deficits are clinically detectable. Interestingly, when the cerebellar-cortical networks are working at a high level, but their collaboration is only partial, as in SCD individuals, a subjective cognitive impairment is perceived.

The positive correlations between network connectivity and MMSE scores across the entire sample underscore the critical role of the cerebellum in maintaining general cognitive efficiency along the AD continuum. The correlation between CBGC network connectivity and CR levels in the AD group emphasizes the sensitivity of this network to reserve effects in accordance with previous studies (Bozzali et al., 2015; Serra et al., 2017). These findings once more support the hypothesis that individuals with higher reserve require greater neuropathological damage before showing cognitive impairment (Stern et al., 2020). Interestingly, the components of the CBGC network appear to be particularly sensitive to CbCR effects. The involvement of the CBGC network is particularly noteworthy, given its established role in executive function, motor control, and cognitive-motor integration. The extensive connectivity of the cerebellum with prefrontal and parietal cortices (Allen et al., 2005; Allen et al., 1997) positions it as a critical node in cognitive networks. Our findings suggest that CbCR may enhance brain capacity to maintain cognitive performance despite alterations in network connectivity.

### 4.4 Study limitations

The cross-sectional design limits our ability to determine whether observed differences reflected protective CR effects or pre-existing individual variations. The unidimensional CR measure (years of education) may not capture the full complexity of reserve mechanisms, and MMSE scores may exhibit ceiling effects, particularly in highly educated populations. Remarkably, the CR effect may be blurred by an intrinsic bias due to the proxy measure used (years of formal education). In fact, older Italian birth cohorts had significantly different access to formal education than younger cohorts due to historical and socioeconomic factors, resulting in an unavoidable unequal distribution of educational level among groups. We are aware that the crossed cerebellar diaschisis and secondary degeneration mechanisms could influence the cerebellar changes within the networks described; unfortunately, the SBM technique does not allow for separating the different regions in the networks for testing the eventual presence of diaschisis effect. Future longitudinal studies with comprehensive biomarker assessment and multidimensional reserve measures are needed to establish causal relationships and track individual trajectories of cognitive decline.

## 5 Conclusion

This study provides evidence for the hypothesis that there is progressive deterioration of cerebellar-cortical GMC connectivity (CBGC, ACSMARC, and PCOC) across the AD continuum, and that there is evidence for the CbCR that modulates network integrity and cognitive function. Notably, the three networks in which the cerebellum was the only common structure exhibited different vulnerability patterns, supporting a dynamic model of cerebellar reserve, wherein effects shift from memory functions in preclinical stages to executive and visuospatial abilities in the most advanced clinical stage of AD. Very recently, it has been suggested that, in parallel with the development of the more advanced AD stage, the loss of cerebellar glomeruli may represent the synaptic mechanism of the cerebellar cognitive reserve (Samstag et al., 2025). These findings highlight the critical cerebellar role in cognitive resilience and suggest that cerebellar reserve assessment and targeted interventions may represent promising approaches for delaying cognitive decline through preserved network integrity.

## Data Availability

The datasets presented in this article are not readily available because for the privacy policy of the institution, the data have to be requested directly to the IRCCS Fondazione Santa Lucia. Requests to access the datasets should be directed to l.serra@hsantalucia.it.

## Footnotes

1
https://trendscenter.org/software/gift/

2
https://www.ibm.com/it-it/analytics/spss-statistics-software
