# Supplementary material for: Bridging cognitive reserve and cerebellar networks: counteracting brain damage in patients with Alzheimer’s disease at different clinical stages
Source: Front Cell Neurosci. 2026 Feb 6;20:1716783. doi: 10.3389/fncel.2026.1716783 (PMC12920246; doi:10.3389/fncel.2026.1716783)
Supplement: Supplementary file 1 [file Table_1.DOCX]

**Supplementary materials**

**1. Demographical and clinical features**

**1.1. Demographical variables on four diagnostic groups**

There were no significant differences between groups in mean age (F_3,175_=3.36, p=0.072). and in sex distribution (AD vs aMCI: Chi^2^= 0.20, df=1, p=0.651; AD vs SCD: Chi^2^= 0.19, df=1, p=0.665; AD vs HS: Chi^2^= 1.30, df=1, p=0.254; aMCI vs SCD: Chi^2^= 0.00, df=1, p=0.993; aMCI vs HS: Chi^2^= 0.55, df=1, p=0.456; SCD vs HS: Chi^2^= 0.54, df=1, p=0.462). As expected, there were significant difference in mean years of formal education (F_3,175_=7.19, p<0.001) and MTA scores (F_3,175_=17.7, p<0.001) because AD patients were less educated and more atrophic in the MTL structure than other groups (until p<0.01 in all comparisons); aMCI patients showed higher MTA scores than SCD and HS groups (always p<0.02). Table 1S shows the demographic features of the four groups.

**Table 1S. Demographic and clinical characteristics**

|  | **AD** | **a-MCI** | **SCD** | **HS** |
| --- | --- | --- | --- | --- |
| **Age (mean+ SD)** | 71.0 +5.4 | 70.5 +7.3 | 68.1 +7.6 | 67.9 + 6.7 |
| **Years of formal education** (mean+ SD) | 10.4 + 4.6* | 12.8 + 4.1* | 14.3 + 7.6* | 14.3 + 3.6* |
| **Sex M/F** | 16/30 | 20/31 | 18/28 | 17/19 |
| **MTA** (mean+ SD) | 2.1 (0.9)*^ | 1.6(1.1)# £ | 0.7 (0.8) | 1.1 (0.8) |

*p<0.05 AD group *vs.* other groups

Abbreviations: AD= Alzheimer’s Disease; a-MCI=amnestic Mild Cognitive Impairment; HS= Healthy Subjects; SCD=Subjective Cognitive Decline; F = Female; M = Male; MTA=Medio Temporal Lobe Scale.

**1.2. Demographical variables on the eight groups divided according to their Cognitive Reserve (CR) level.**

There were no significant differences in the mean age comparing participants with L_CR_ against those with H_CR_ (F_7,171_=2.06, p=0.06). there were no significant differences in sex distribution (AD-L_CR_ vs AD-H_CR_: Chi^2^= 0.16, df=1, p=0.686; AD-L_CR_ vs aMCI-L_CR_: Chi^2^= 0.22, df=1, p=0.641; AD-L_CR_ vs aMCI-H_CR_: Chi^2^= 1.02, df=1, p=0.312; AD-L_CR_ vs SCD-L_CR_: Chi^2^= 0.13, df=1, p=0.716; AD-L_CR_ vs SCD-H_CR_: Chi^2^= 0.57, df=1, p=0.451; AD-L_CR_ vs HS-L_CR_: Chi^2^= 0.47, df=1, p=0.495; AD-L_CR_ vs HS-H_CR_: Chi^2^= 1.77, df=1, p=0.183; AD-H_CR_ vs aMCI-L_CR_: Chi^2^= 0.01, df=1, p=0.926; AD-H_CR_ vs aMCI-H_CR_: Chi^2^= 0.36, df=1, p=0.548; AD-H_CR_ vs SCD-L_CR_: Chi^2^= 0.00, df=1, p=0.950; AD-H_CR_ vs SCD-H_CR_: Chi^2^= 0.16, df=1, p=0.692; AD-H_CR_ vs HS-L_CR_: Chi^2^= 0.09, df=1, p=0.759; AD-H_CR_ vs HS-H_CR_: Chi^2^= 0.96, df=1, p=0.326; aMCI-L_CR_ vs aMCI-H_CR_: Chi^2^= 0.20, df=1, p=0.651; aMCI-L_CR_ vs SCD-L_CR_: Chi^2^= 0.02, df=1, p=0.877; aMCI-L_CR_ vs SCD-H_CR_: Chi^2^= 0.08, df=1, p=0.773; aMCI-L_CR_ vs HS-L_CR_: Chi^2^= 0.04, df=1, p=0.842; aMCI-L_CR_ vs HS-H_CR_: Chi^2^= 0.7, df=1, p=0.404; aMCI-H_CR_ vs SCD-L_CR_: Chi^2^= 0.50, df=1, p=0.479; aMCI-H_CR_ vs SCD-H_CR_: Chi^2^= 0.01, df=1, p=0.910; aMCI-H_CR_ vs HS-L_CR_: Chi^2^= 0.05, df=1, p=0.814; aMCI-H_CR_ vs HS-H_CR_: Chi^2^= 0.25, df=1, p=0.615; SCD-L_CR_ vs SCD-H_CR_: Chi^2^= 0.22, df=1, p=0.639; SCD-L_CR_ vs HS-L_CR_: Chi^2^= 0.14, df=1, p=0.703; SCD-L_CR_ vs HS-H_CR_: Chi^2^= 0.18, df=1, p=0.278; SCD-H_CR_ vs HS-L_CR_: Chi^2^= 0.01, df=1, p=0.922; SCD-H_CR_ vs HS-H_CR_: Chi^2^= 0.28, df=1, p=0.597; HS-L_CR_ vs HS-H_CR_: Chi^2^= 0.42, df=1, p=0.515. Obviously, there were significant differences in years of formal education (F_7,171_=90.14, p<0.0001). Namely, AD-L_CR_ groups showed significant differences compared to all groups (p<0.0001), except in comparison to aMCI-L_CR_ group (p=0.125); patients with AD-H_CR_ showed significant differences compared to all groups (p<0.05), except in contrast to aMCI-H_CR_ group (p=0.885); patients with aMCI-L_CR_ showed significant differences compared to all groups (p<0.0001), except in comparison to AD-L_CR_ (p=0.125); patients with aMCI-H_CR_ showed significant differences compared to all groups (p<0.01), except in comparison to AD-H_CR_ (p=0.885, as reported above) and to SCD-H_CR_ (p=0.273); individuals with SCD-L_CR_ were significantly different from all groups (p<0.0001), with the exception of HS-L_CR_ (p=0.996); individuals with SCD-H_CR_ were significantly different from all groups (p<0.05) with the exception of aMCI-H_CR_ (p=0.273) and HS-H_CR_ (p=0.969) groups; HS-L_CR_ group was significantly different from all groups (p<0.0001), with the exception of SCD-L_CR_ (p=0.996) group; HS-H_CR_ group was significantly different from all groups (p<0.01), with the exception of SCD-H_CR_ (p=0.969) group.

1. **Neuropsychological results**
   1. ***Diagnostic groups:***

As expected, the four diagnostic groups differed in both measures of cognitive efficiency, MMSE scores (F_3,175_=98.1, p<0.00001) and ACE-R total scores (F_3,175_=170.8, p<0.00001). In both tests, AD patients scored worse than all other groups (p<0.00001 in each comparison). aMCI patients obtained lower scores than SCD and HS groups (p<0.00001 in each comparison). No significant differences between SCD individuals and HS were observed in general cognitive performance (p=0.997 for both MMSE and ACE-R total scores).

Several statistically significant differences were found in the ACE-R domains, including Orientation/Attention (F_3,175_=58.5, p<0.00001), Memory (F_3,175_=145.2, p<0.00001), Executive Functions (F_3,175_=56.3, p<0.00001), Language domain (F_3,175_=55.9, p<0.00001), and Visuo-Spatial abilities domain (F_3,175_=21.7, p<0.00001). In particular, AD and aMCI patients showed lower performance than other groups across all cognitive domains (p<0.05 in each analysis), while no significant differences were observed between SCD individuals and HS.

- 1. ***Groups divided according to CR levels***

**Table 2S: A) MMSE**

|  | MMSE (F_7,171_=38.6, p<0.0001) **Post-hoc** | | | | | | | |
| --- | --- | --- | --- | --- | --- | --- | --- | --- |
|  | **AD-L_CR_**  p-level: | **AD-H_CR_**  p-level: | **aMCI-L_CR_**  p-level: | **aMCI-H_CR_**  p-level: | **SCD-L_CR_**  p-level: | **SCD-H_CR_**  p-level: | **HS-L_CR_**  p-level: | **HS-H_CR_**  p-level: |
| **AD-L_CR_** | - | 0.999 | **0.0001** | **0.0001** | **0.0001** | **0.0001** | **0.0001** | **0.0001** |
| **AD-H_CR_** | 0.999 | - | **0.0001** | **0.0001** | **0.0001** | **0.0001** | **0.0001** | **0.0001** |
| **aMCI-L_CR_** | **0.0001** | **0.0001** | - | 0.999 | **0.0001** | **0.002** | **0.001** | **0.001** |
| **aMCI-H_CR_** | **0.0001** | **0.0001** | 0.999 | - | **0.0001** | **0.002** | **0.0001** | **0.001** |
| **SCD-L_CR_** | **0.0001** | **0.0001** | **0.0001** | **0.0001** | - | 1 | 1 | 1 |
| **SCD-H_CR_** | **0.0001** | **0.0001** | **0.002** | **0.002** | 1 | - | 1 | 1 |
| **HS-L_CR_** | **0.0001** | **0.0001** | **0.001** | **0.0001** | 1 | 1 | - | 1 |
| **HS-H_CR_** | **0.0001** | **0.0001** | **0.001** | **0.001** | 1 | 1 | 1 | - |

**Table 2S: B) ACE-R total**

|  | **ACE-R Total (**F_7,171_=62.9, p<0.0001) **Post-hoc** | | | | | | | |
| --- | --- | --- | --- | --- | --- | --- | --- | --- |
|  | **AD-L_CR_**  p-level: | **AD-H_CR_**  p-level: | **aMCI-L_CR_**  p-level: | **aMCI-H_CR_**  p-level: | **SCD-L_CR_**  p-level: | **SCD-H_CR_**  p-level: | **HS-L_CR_**  p-level: | **HS-H_CR_**  p-level: |
| **AD-L_CR_** | - | 1 | 0.0001 | 0.0001 | 0.0001 | 0.0001 | 0.0001 | 0.0001 |
| **AD-H_CR_** | 1 | - | 0.0001 | 0.0001 | 0.0001 | 0.0001 | 0.0001 | 0.0001 |
| **aMCI-L_CR_** | 0.0001 | 0.0001 | - | 0.785 | 0.0001 | 0.0001 | 0.0001 | 0.0001 |
| **aMCI-H_CR_** | 0.0001 | 0.0001 | 0.785 | - | 0.001 | 0.007 | 0.001 | 0.001 |
| **SCD-L_CR_** | 0.0001 | 0.0001 | 0.0001 | 0.001 | - | 1 | 1 | 0.895 |
| **SCD-H_CR_** | 0.0001 | 0.0001 | 0.0001 | 0.007 | 1 | - | 1 | 0.975 |
| **HS-L_CR_** | 0.0001 | 0.0001 | 0.0001 | 0.001 | 1 | 1 | - | 0.994 |
| **HS-H_CR_** | 0.0001 | 0.0001 | 0.0001 | 0.0001 | 0.895 | 0.975 | 0.994 | - |

**Table 2S: C) Orientation/Attention**

|  | Orientation/Attention (F_7,171_=24.4, p<0.0001) **Post-hoc** | | | | | | | |
| --- | --- | --- | --- | --- | --- | --- | --- | --- |
|  | **AD-L_CR_**  p-level: | **AD-H_CR_**  p-level: | **aMCI-L_CR_**  p-level: | **aMCI-H_CR_**  p-level: | **SCD-L_CR_**  p-level: | **SCD-H_CR_**  p-level: | **HS-L_CR_**  p-level: | **HS-H_CR_**  p-level: |
| **AD-L_CR_** | - | 0.764 | 0.0001 | 0.0001 | 0.0001 | 0.0001 | 0.0001 | 0.0001 |
| **AD-H_CR_** | 0.764 | - | 0.0001 | 0.0001 | 0.0001 | 0.0001 | 0.0001 | 0.0001 |
| **aMCI-L_CR_** | 0.0001 | 0.0001 | - | 1 | 0.130 | 0.300 | 0.240 | 0.347 |
| **aMCI-H_CR_** | 0.0001 | 0.0001 | 1 | - | 0.037 | 0.175 | 0.185 | 0.202 |
| **SCD-L_CR_** | 0.0001 | 0.0001 | 0.130 | 0.003 | - | 1 | 1 | 1 |
| **SCD-H_CR_** | 0.0001 | 0.0001 | 0.307 | 0.175 | 1 | - | 1 | 1 |
| **HS-L_CR_** | 0.0001 | 0.0001 | 0.244 | 0.118 | 1 | 1 | - | 1 |
| **HS-H_CR_** | 0.0001 | 0.0001 | 0.347 | 0.202 | 1 | 1 | 1 | - |

**Table 2S: D) Memory**

|  | Memory (F_7,171_=56.5, p<0.0001) **Post-hoc** | | | | | | | |
| --- | --- | --- | --- | --- | --- | --- | --- | --- |
|  | **AD-L_CR_**  p-level: | **AD-H_CR_**  p-level: | **aMCI-L_CR_**  p-level: | **aMCI-H_CR_**  p-level: | **SCD-L_CR_**  p-level: | **SCD-H_CR_**  p-level: | **HS-L_CR_**  p-level: | **HS-H_CR_**  p-level: |
| **AD-L_CR_** | - | 1 | 0.0001 | 0.0001 | 0.0001 | 0.0001 | 0.0001 | 0.0001 |
| **AD-H_CR_** | 1 | - | 0.0001 | 0.0001 | 0.0001 | 0.0001 | 0.0001 | 0.0001 |
| **aMCI-L_CR_** | 0.0001 | 0.0001 | - | 0.882 | 0.0001 | 0.0001 | 0.0001 | 0.0001 |
| **aMCI-H_CR_** | 0.0001 | 0.0001 | 0.882 | - | 0.0001 | 0.0001 | 0.0001 | 0.0001 |
| **SCD-L_CR_** | 0.0001 | 0.0001 | 0.0001 | 0.0001 | - | 1 | 1 | 0.856 |
| **SCD-H_CR_** | 0.0001 | 0.0001 | 0.0001 | 0.0001 | 1 | - | 1 | 0.939 |
| **HS-L_CR_** | 0.0001 | 0.0001 | 0.0001 | 0.0001 | 1 | 1 | - | 0.986 |
| **HS-H_CR_** | 0.0001 | 0.0001 | 0.0001 | 0.0001 | 0.856 | 0.939 | 0.986 | - |

**Table 2S: E) Executive functions**

|  | Executive Functions (F_7,171_=21.7, p<0.0001) **Post-hoc** | | | | | | | |
| --- | --- | --- | --- | --- | --- | --- | --- | --- |
|  | **AD-L_CR_**  p-level: | **AD-H_CR_**  p-level: | **aMCI-L_CR_**  p-level: | **aMCI-H_CR_**  p-level: | **SCD-L_CR_**  p-level: | **SCD-H_CR_**  p-level: | **HS-L_CR_**  p-level: | **HS-H_CR_**  p-level: |
| **AD-L_CR_** | - | 1 | 0.037 | 0.0001 | 0.0001 | 0.0001 | 0.0001 | 0.0001 |
| **AD-H_CR_** | 1 | - | 0.026 | 0.0001 | 0.0001 | 0.0001 | 0.0001 | 0.0001 |
| **aMCI-L_CR_** | 0.037 | 0.026 | - | 0.205 | 0.036 | 0.013 | 0.003 | 0.001 |
| **aMCI-H_CR_** | 0.0001 | 0.0001 | 0.205 | - | 0.989 | 0.773 | 0.509 | 0.250 |
| **SCD-L_CR_** | 0.0001 | 0.0001 | 0.036 | 0.989 | - | 0.991 | 0.935 | 0.726 |
| **SCD-H_CR_** | 0.0001 | 0.0001 | 0.013 | 0.773 | 0.991 | - | 1 | 0.997 |
| **HS-L_CR_** | 0.0001 | 0.0001 | 0.003 | 0.509 | 0.935 | 1 | - | 1 |
| **HS-H_CR_** | 0.0001 | 0.0001 | 0.001 | 0.250 | 0.726 | 0.997 | 1 | - |

**Table 2S: F) Language**

|  | **Language** (F_7,171_=23.1, p<0.0001) **Post-hoc** | | | | | | | |
| --- | --- | --- | --- | --- | --- | --- | --- | --- |
|  | **AD-L_CR_**  p-level: | **AD-H_CR_**  p-level: | **aMCI-L_CR_**  p-level: | **aMCI-H_CR_**  p-level: | **SCD-L_CR_**  p-level: | **SCD-H_CR_**  p-level: | **HS-L_CR_**  p-level: | **HS-H_CR_**  p-level: |
| **AD-L_CR_** | - | 0.282 | 0.0001 | 0.0001 | 0.0001 | 0.0001 | 0.0001 | 0.0001 |
| **AD-H_CR_** | 0.282 | - | 0.0001 | 0.0001 | 0.0001 | 0.0001 | 0.0001 | 0.0001 |
| **aMCI-L_CR_** | 0.0001 | 0.0001 | - | 1 | 0.957 | 0.956 | 0.975 | 0.872 |
| **aMCI-H_CR_** | 0.0001 | 0.0001 | 1 | - | 0.560 | 0.666 | 0.717 | 0.452 |
| **SCD-L_CR_** | 0.0001 | 0.0001 | 0.957 | 0.560 | - | 1 | 1 | 1 |
| **SCD-H_CR_** | 0.0001 | 0.0001 | 0.956 | 0.666 | 1 | - | 1 | 1 |
| **HS-L_CR_** | 0.0001 | 0.0001 | 0.975 | 0.717 | 1 | 1 | - | 1 |
| **HS-H_CR_** | 0.0001 | 0.0001 | 0.872 | 0.452 | 1 | 1 | 1 | - |

**Table 2S: G) Visuo-spatial abilities**

|  | Visuo-spatial abilities (F_7,171_=10.4, p<0.0001) **Post-hoc** | | | | | | | |
| --- | --- | --- | --- | --- | --- | --- | --- | --- |
|  | **AD-L_CR_**  p-level: | **AD-H_CR_**  p-level: | **aMCI-L_CR_**  p-level: | **aMCI-H_CR_**  p-level: | **SCD-L_CR_**  p-level: | **SCD-H_CR_**  p-level: | **HS-L_CR_**  p-level: | **HS-H_CR_**  p-level: |
| **AD-L_CR_** | - | 0.080 | 0.0001 | 0.0001 | 0.0001 | 0.0001 | 0.0001 | 0.0001 |
| **AD-H_CR_** | 0.080 | - | 0.365 | 0.195 | 0.007 | 0.014 | 0.043 | 0.007 |
| **aMCI-L_CR_** | 0.0001 | 0.365 | - | 1 | 0.957 | 0.902 | 0.991 | 0.840 |
| **aMCI-H_CR_** | 0.0001 | 0.195 | 1 | - | 0.890 | 0.820 | 0.977 | 0.722 |
| **SCD-L_CR_** | 0.0001 | 0.007 | 0.957 | 0.890 | - | 1 | 1 | 1 |
| **SCD-H_CR_** | 0.0001 | 0.014 | 0.902 | 0.820 | 1 | - | 1 | 1 |
| **HS-L_CR_** | 0.0001 | 0.043 | 0.991 | 0.977 | 1 | 1 | - | 0.998 |
| **HS-H_CR_** | 0.0001 | 0.007 | 0.840 | 0.722 | 1 | 1 | 0.998 | - |

**Supplementary Figure Legend.** **ICASSO Validation of Structural Network Robustness**. This figure displays the results of the ICASSO (Independent Component Analysis by Stability SPlitting) procedure, validating the stability and reproducibility of the Independent Components (ICs) extracted via Source-Based Morphometry (SBM). The validation process is founded upon the Similarity Matrix, which quantifies the pairwise correlation among IC estimates obtained from *N* repeated (bootstrapped) ICA runs. The clustering results are visualized through the Estimate quality, Similarity Graph and the Dendrogram. High coherence within each component is demonstrated by the formation of tight, well-separated clusters in the dendrogram, which translates to a high Clustering Quality Index (Iq). This robust clustering demonstrates that the derived structural network patterns (ICs) and their associated Source Estimates (or mixing coefficients) are highly reliable and invariant to changes in algorithm initialization. This high degree of stability provides strong confidence in subsequent statistical analyses performed on the subject-specific expression of these networks. The components corresponding to the Cerebellum-Basal Ganglia-Cingulum (CBGC) network (Component 1), the Anterior Cerebellum-Supplementary Motor Area-Retrosplenial Cortex (ACSMARC) network (Component 4), and the Posterior Cerebellum-Orbitofrontal Cortex (PCOC) network (Component 10) are highlighted by red circles.
