# Supplementary figures and images for: Bridging cognitive reserve and cerebellar networks: counteracting brain damage in patients with Alzheimer’s disease at different clinical stages
Source: Front Cell Neurosci. 2026 Feb 6;20:1716783. doi: 10.3389/fncel.2026.1716783 (PMC12920246; doi:10.3389/fncel.2026.1716783)

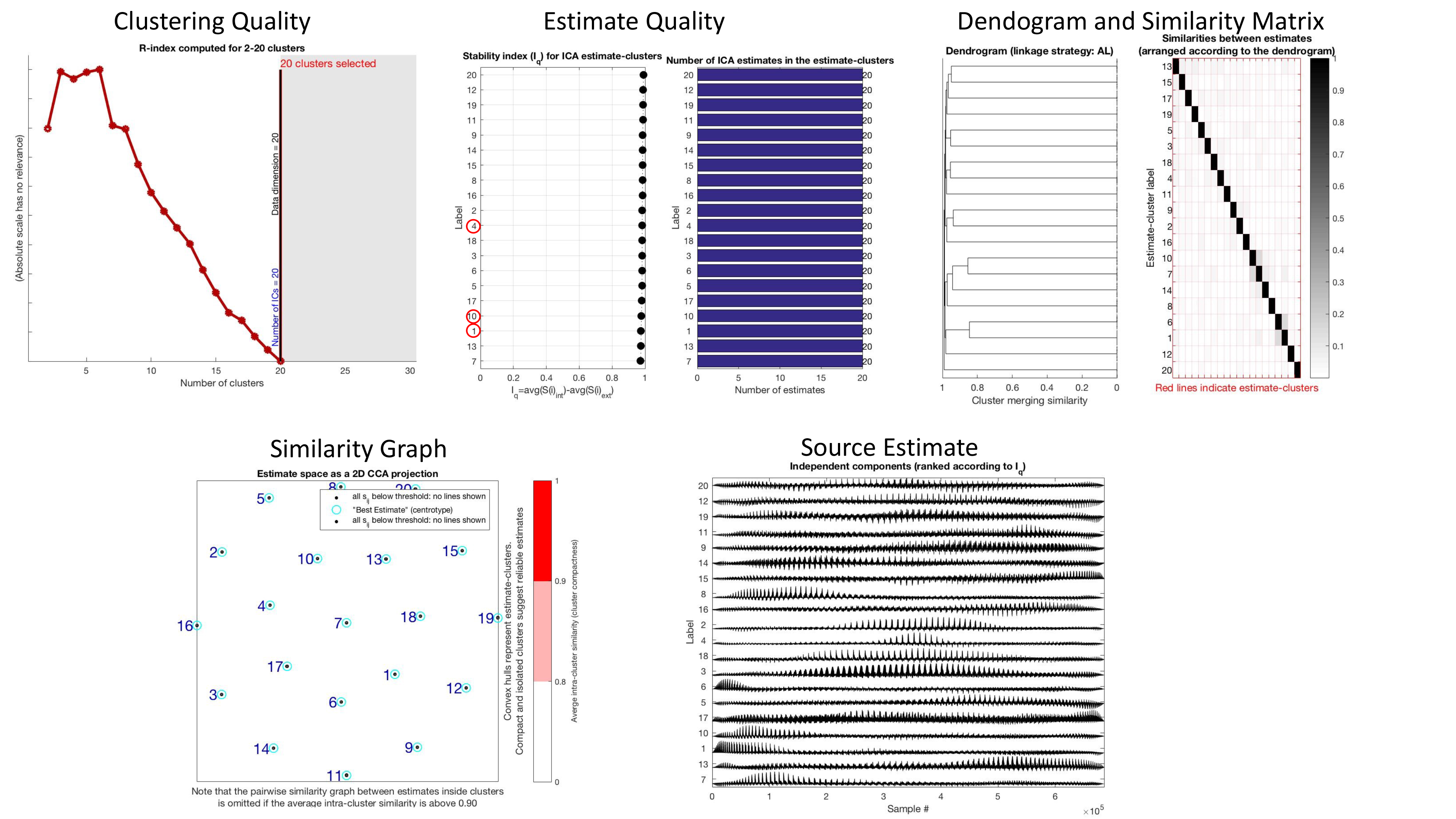

Supplement: Supplementary file 2 [file Image_1.TIF]
